# Assembly and dynamic regulation of the tip filament of the *Bordetella* type III secretion system injectisome

**DOI:** 10.1128/mbio.01135-25

**Published:** 2025-09-22

**Authors:** Ivana Malcova, Martin Zmuda, Jan Valecka, Jana Kamanova

**Affiliations:** 1 Laboratory of Infection Biology, Institute of Microbiology of the Czech Academy of Sciences, Videnska 86863, Prague, Czechia; 2 Light Microscopy Core Facility, Institute of Molecular Genetics of the Czech Academy of Sciences, Videnska, Prague, Czechia; University of Texas Health Science Center, School of Public Health, Houston, Texas, USA

**Keywords:** *Bordetella*, type III secretion system, needle tip filament, tip protein, Bsp22, EspA

## Abstract

Many gram-negative bacteria utilize a type III secretion system (T3SS) injectisome to translocate effector proteins directly into host cells. In *Bordetella bronchiseptica*, a respiratory pathogen of diverse mammals, the T3SS injectisome contains a unique needle tip filament formed by the Bsp22 protein, which is essential for bacterial persistence in mice. Here, we used *B. bronchiseptica* and *Bordetella pertussis* strains with in-frame insertions of peptide tags into Bsp22 to investigate filament formation using super-resolution imaging with fluorophore-labeled nanobodies and biarsenic probes. During cultivation on glass coverslips, more than 50% of the bacteria had flexible Bsp22 filaments with an average length of 1 µm after 3 hours of incubation. Filament growth occurred continuously at the distal end. In contrast, during HeLa cell infection, although the T3SS effector BteA was delivered into host cells and the number of filaments per bacterium remained unchanged, filament length was significantly shorter, averaging 0.4 µm after 3 hours of infection. Some Bsp22 filaments formed short physical bridges between bacteria and host cells. During infection of nasal epithelial cells in air-liquid interface cultures, Bsp22 filaments became scarce, with most bacteria lacking detectable filaments. This reduction correlated with a specific decline in *bsp22* mRNA levels, while mRNA levels of *bscD*, encoding the inner membrane ring component of the injectisome, remained stable. Our findings demonstrate that Bsp22 synthesis is tightly regulated in response to environmental cues and highlight how filament characteristics of *Bordetella* T3SS injectisome change in different environments.

**IMPORTANCE:**

*Bordetella bronchiseptica* and *Bordetella pertussis* are two closely related respiratory pathogens that employ their T3SS injectisome to deliver the BteA effector into host cells. In this study, we visualized the needle tip filament of their T3SS injectisome, a structure formed by the Bsp22 protein. We demonstrate that during *Bordetella* cultivation in Stainer-Scholte medium, Bsp22 filaments are abundant and can dynamically extend up to several micrometers in length through the incorporation of new subunits at their distal ends. In contrast, these filaments become shorter and/or less abundant during infection of host cells. This reduction correlates with decreased *bsp22* mRNA expression and lower Bsp22 protein levels, while the levels of *bscD* mRNA, which encodes the inner membrane ring protein of the injectisome, remain stable. These results highlight the adaptability of the *Bordetella* T3SS injectisome and show how its tip filament structure changes in response to different environments.

## INTRODUCTION

The classical *Bordetella* species, specifically *B. bronchiseptica*, *B. pertussis*, and *B. parapertussis*, are respiratory pathogens that colonize the ciliated epithelial cells of the mammalian respiratory tract. *B. bronchiseptica* infects a variety of mammals, leading to diverse pathologies. These include persistent, often asymptomatic respiratory infections, as well as acute diseases such as kennel cough in dogs and bronchopneumonia and atrophic rhinitis in piglets (1). The strictly human-adapted *B. pertussis* and the human-adapted lineage of *B. parapertussis*_HU_, on the other hand, are responsible for causing whooping cough, also known as pertussis, which remains a significant concern for public health and one of the least controlled vaccine-preventable infectious diseases (2–4). It is believed that *B. pertussis* and *B. parapertussis*_HU_ evolved independently from a *B. bronchiseptica*-like ancestor, sharing highly identical genetic content, including genes that encode the type III secretion system (T3SS) injectisomes (5–7).

The T3SS injectisomes function as syringe-like nanomachines embedded in the bacterial envelope, facilitating the direct translocation of effector proteins from the bacterial cytosol into the host cell cytoplasm. The translocation process involves recruiting secreted effector proteins to the cytoplasmic sorting platform, loading them into the inner membrane export apparatus, and subsequently transporting them through a continuous channel within the injectisome basal body and the extracellular needle filament. The needle filament is capped by a tip complex that connects it to the translocon pore in the host cell membrane, thus creating a complete channel (8, 9). These nanomachines likely arose by exaptation of the bacterial flagellum, which was followed by their rapid diversification and adaptation to different host cells and bacterial lifestyles (10, 11).

The prototypical injectisome, as encoded in pathogens such as *Salmonella*, *Shigella*, and *Yersinia*, is characterized by a needle filament measuring 45–80 nm in length, capped by a ring-shaped pentameric tip complex composed of the tip protein (12–16). Interestingly, in attaching and effacing pathogens (A/E pathogens), comprising enteropathogenic *Escherichia coli* (EPEC), this pentameric tip complex is replaced by a tip filament, an extended structure connected to the needle, formed through the polymerization of a tip protein EspA (17–20). Similarly, in *B. bronchiseptica*, immunoelectron microscopy has shown that a tip protein Bsp22 forms surface appendages (21). Due to the absence of significant sequence similarity to EspA and the pentameric T3SS-tip complex protein families SipD/IpaD (*Salmonella* and *Shigella* spp.) and LcrV/PcrV (*Yersinia* and *Pseudomonas* spp.), it has been proposed that Bsp22 defines a novel T3SS-tip complex family (21, 22).

Bsp22 is the most abundantly secreted T3SS substrate of *B. bronchiseptica* during cultivation in Stainer-Scholte medium (SSM). Its role in the *Bordetella* injectisome tip complex is evidenced by the fact that Bsp22 inactivation does not block T3SS-mediated secretion into the medium but does block the translocation of the cytotoxic BteA effector into host cells, thereby hindering T3SS-mediated cytotoxicity (21, 23). Additionally, similar to *Bordetella* T3SS ATPase BscN, Bsp22 is crucial for the persistence of *B. bronchiseptica* in mouse trachea (24). Moreover, while immunization of mice with Bsp22 does not confer protection against an intranasal challenge with *B. pertussis*, it does lead to a significant reduction in colonization by *B. bronchiseptica* (21, 25).

Although the significance of Bsp22 filaments is recognized, our understanding of their formation and dynamics during *Bordetella* infection remains limited, particularly in relation to their potential similarities and differences compared to EspA filaments of A/E pathogens. Therefore, the aim of this study was to visualize Bsp22 filaments both on glass coverslips and during host cell infection using high-resolution fluorescence imaging. This investigation is critical for elucidating the mechanism of action of the T3SS injectisome in classical *Bordetella* species. It also contributes to a broader understanding of the assembly and functional diversity of T3SS injectisome families across different bacterial pathogens.

## RESULTS

### The Bsp22 protein polymerizes into flexible filaments that intertwine on the surface of a glass coverslip

**Fig 1:**
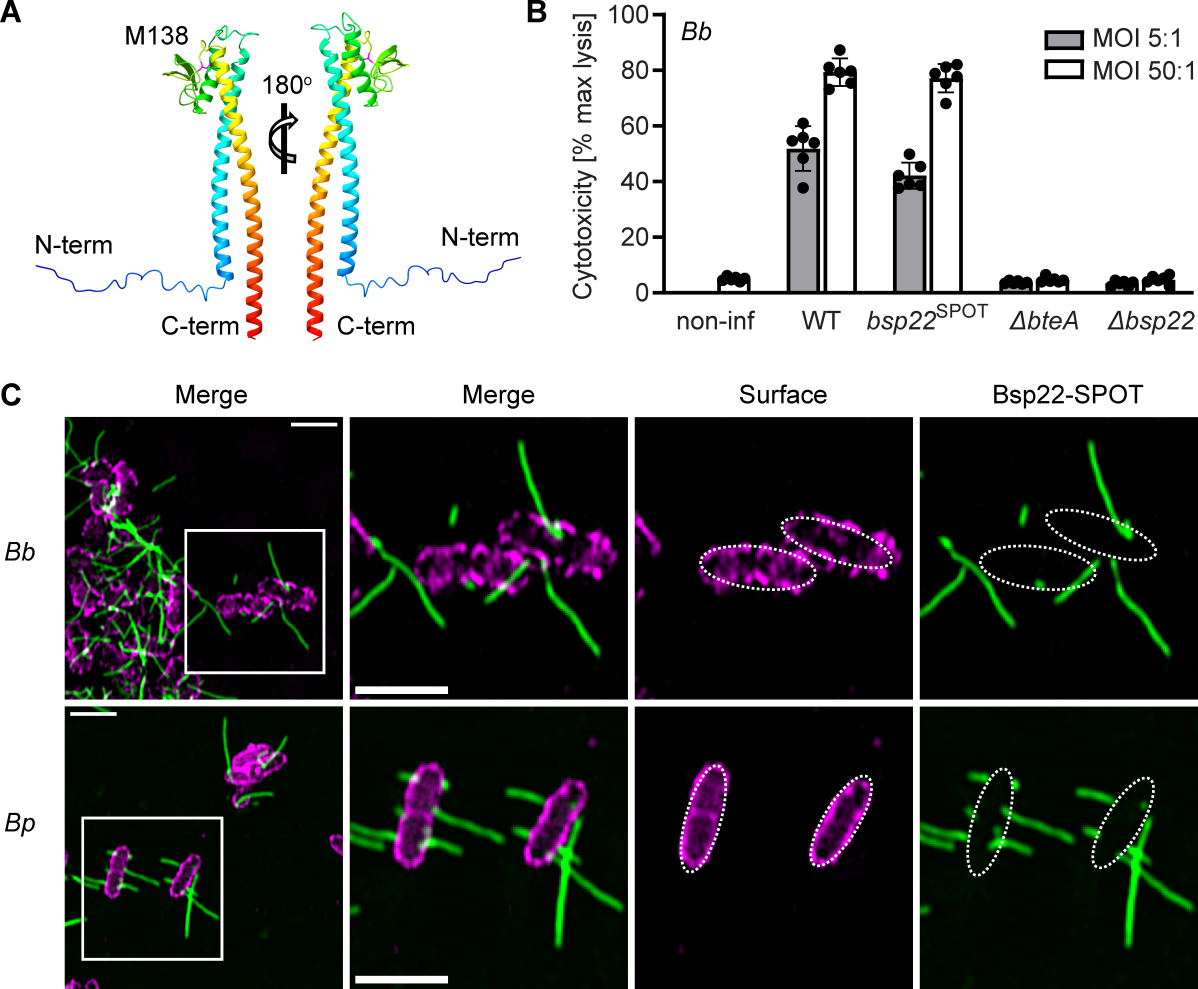
The Bsp22 protein polymerizes into flexible filaments that intertwine across the surface of a glass coverslip. (**A**) Ribbon representation of the AlphaFold-predicted monomeric structure of Bsp22 from *B. bronchiseptica* RB50. The unstructured loop of the mixed α/β region is highlighted, showing the position of the M138 residue. This representation was generated by UCSF ChimeraX. (**B**) Insertion of a SPOT-tag between amino acids M138 and A139 of Bsp22 does not affect the functionality of the injectisome. T3SS-dependent cytotoxicity of the indicated *B. bronchiseptica* strains toward HeLa cells was determined using a LDH release assay at 3 hours post-infection. Data represent the mean ± SD from two independent biological experiments, each with three technical replicates. (**C**) Bsp22 filaments are formed on the surface of *B. bronchiseptica* and *B. pertussis*. Cells of *B. bronchiseptica* RB50 (*Bb*) or *B. pertussis* B1917 (*Bp*) carrying *bsp22*^SPOT^ were cultured on coverslips in *Bb*-SSM or *Bp*-SSM, respectively, fixed at 3 hours and stained. Bsp22, green; bacterial cell surface, magenta. The SIM images show a single focal plane and are representative of three independent experiments. Scale bars, 2 µm.

To enable super-resolution fluorescence imaging, we first investigated whether Bsp22 would tolerate the insertion of a short peptide SPOT-tag recognized by a commercially available nanobody without affecting Bsp22 function in the *Bordetella* injectisome. To select a suitable insertion site, we examined the three-dimensional structure of Bsp22 from *B. bronchiseptica* RB50 predicted by AlphaFold. The structure revealed a long N-terminal α-helix (I26-A74) connected to a C-terminal α-helix (Y144-M204) by a mixed α/β-region, with a surface-exposed unstructured loop (T135–T143) (Fig. 1A). Given that a similar surface-exposed loop in the EspA protein was previously demonstrated to tolerate insertions (26, 27), we opted to insert the SPOT-tag flanked by GSSG linkers between amino acid positions M138 and A139 of Bsp22, resulting in a *B. bronchiseptica* RB50 derivative, *Bb bsp22*^SPOT^. To assess the injectisome functionality of the *Bb bsp22*^SPOT^, we examined its ability to induce T3SS-dependent cell cytotoxicity mediated by injection of the type III effector protein BteA. As shown in Fig. 1B, *Bb bsp22*^SPOT^ exhibited cell cytotoxicity comparable to that of the wild-type strain. After a 3-hour infection, both strains induced lysis of 50% and 80% of HeLa cells at a multiplicity of infection (MOI) of 5:1 and 50:1, respectively. In contrast, infection with RB50 derivatives harboring in-frame deletions of the open reading frames for Bsp22 (*Bb* Δ*bsp22*) or the type III effector BteA (*Bb* Δ*bteA*) did not induce cell lysis. These data demonstrate that the Bsp22 protein can tolerate the insertion of a short peptide tag between amino acids M138 and A139.

Next, we employed structured illumination microscopy (SIM) to examine Bsp22 appendages in the *Bb bsp22^SPOT^* strain. To facilitate visualization, cells of *Bb bsp22^SPOT^* were diluted in *Bb*-SSM cultivation medium (Table S1), distributed into 6-well plates containing uncoated high-precision glass coverslips and centrifuged to promote their attachment to coverslips. The plates were then incubated for 3 hours, after which cells were fixed and Bsp22 was labeled using an anti-SPOT nanobody conjugated to ATTO488, Spot-label ATTO488. As shown in Fig. 1C; Fig. S1, Bsp22 polymerized into filaments that extended in different directions from *B. bronchiseptica* cells and had varying lengths. These filaments were found in very low numbers per bacterial cell, ranging from none to a few, with some reaching lengths of over two μm. The filaments rarely protruded from cell poles. We also observed that these filaments intertwine and twist, indicating their flexibility. Importantly, similar Bsp22 filaments of different lengths, twisting in different directions, were also produced by *B. pertussis* B1917 derivative, *Bp bsp22*^SPOT^, in which we introduced the SPOT-tag at the same position within Bsp22, that is, in between amino acids M138 and A139 (Fig. 1C). Thus, both classical *Bordetella* species, *B. bronchiseptica*, which causes chronic respiratory infections in a variety of mammals, and *B. pertussis*, an exclusively human pathogen causing whooping cough, produce flexible Bsp22 filaments of heterogeneous length that intertwine on glass coverslips during bacterial growth. Additionally, Bsp22 tolerates the insertion of short peptide tags, allowing for the peptide display on the surface of Bsp22 filaments.

### Growth of Bsp22 filaments is continuous, with Bsp22 monomers being added at the distal filament end

**Fig 2:**
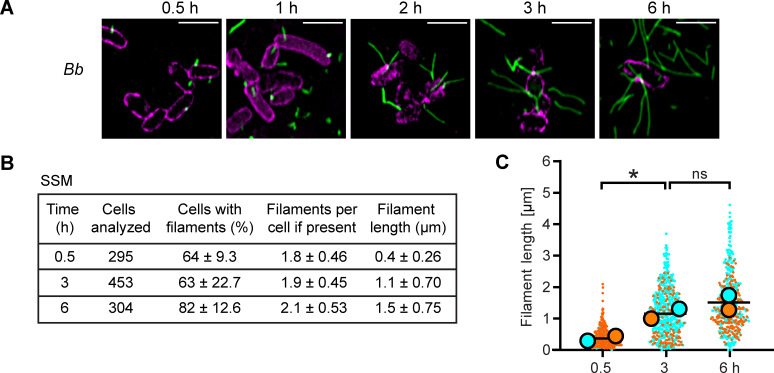
The Bsp22 filaments elongate on glass coverslips. (**A**) Visualization of Bsp22 filaments at different time points. Cells of *Bb bsp22*^SPOT^ were incubated on coverslips in *Bb*-SSM, fixed at indicated time points and stained. Bsp22, green; bacterial cell surface, magenta. Images are single focal planes and representative of three independent experiments. Scale bars, 2 µm. (**B–C**) Quantitative analysis of Bsp22 filaments at different time points. (**B**) The percentage of *Bb bsp22*^SPOT^ cells with filaments, filament count per cell, and filament length. Results are presented as the mean ± SD, calculated from multiple microscopy fields of two independent experiments. (**C**) SuperPlots of filament lengths from two independent experiments. Distinct colors represent each experiment, with circles indicating the mean filament length of each experiment. The black bar shows the overall average. *P < 0.05, unpaired two-tailed *t*-test.

When visualizing Bsp22, we observed filaments of different lengths, suggesting a lack of growth control during their assembly. To analyze the dynamics of filament formation, we cultured *Bb bsp22*^SPOT^ on glass coverslips in bacterial cultivation medium *Bb*-SSM and fixed them at different time points: 0.5, 1, 2, 3, and 6 hours of incubation. When the bacteria were fixed only 30 minutes after spotting, they exhibited short filaments. As the incubation progressed, the length of Bsp22 filaments increased, and by 6 hours post-spotting, these filaments formed a cohesive network (Fig. 2A; Fig. S2). We also noticed some filaments that were torn from the bacterial surface, which was likely due to shaking of the initial bacterial culture or shear force caused by pipetting when diluting the bacterial culture (Fig. 2A; Fig. S2).

To quantify filament growth and abundance, we developed a custom script for FIJI (28) that enables accurate measurement of overlapping and intersecting filaments. This script employs segmentation of *Bordetella* cells and manual tracing of individual Bsp22 filaments (see Materials and Methods). Our analysis showed that the mean filament length increased with incubation time, while the filament count per cell remained constant at an average of two (Fig. 2B). Plots of filament lengths from independent experiments revealed high heterogeneity in Bsp22 filaments lengths within the individual cell population, as well as consistent increases in the mean filament length as incubation progressed (Fig. 2C). A similar increase in the mean filament length was observed for *Bp bsp22*^SPOT^ cells incubated on glass coverslips in *Bp*-SSM (Fig. S3). Quantifications in Fig. 2B; Fig. S3 indicate that the average number of filaments per cell is comparable between *B. bronchiseptica* and *B. pertussis*; however, the filament elongation appears slower in *B. pertussis*. Collectively, these data demonstrate that Bsp22 filaments in both species grow continuously in bacterial cultivation medium.

**Fig 3:**
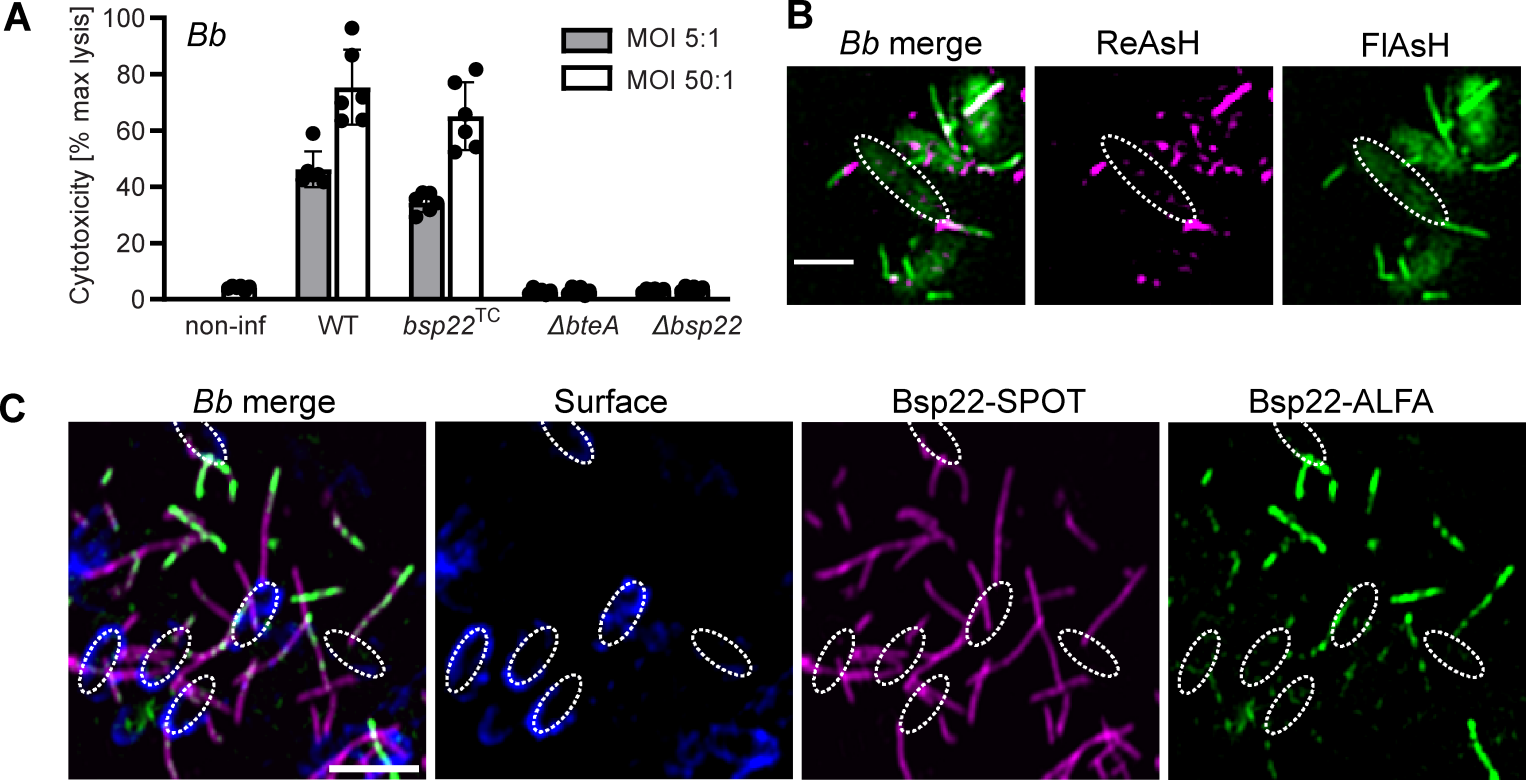
Bsp22 monomers are added at the distal filament end. (**A**) The functionality of the injectisome is not affected by tagging Bsp22 with TC-tag. T3SS-dependent cytotoxicity of the indicated *B. bronchiseptica* strains toward HeLa cells was measured as described in the legend of Fig. 1B. Data represent the mean ± SD from two independent biological experiments, each with three technical replicates. (**B–C**) Bsp22 monomers are added at the distal filament end. In **B**, cells of *Bb bsp22*^TC^ were sequentially labeled with ReAsH (magenta) followed by FlAsH (green) in a pulse-chase experiment. In **C**, cells of *Bb bsp22*^SPOT^ carrying a plasmid for inducible expression of Bsp22^ALFA^ were seeded on coverslips in *Bb*-SSM. After 3 hours, Bsp22^ALFA^ expression was induced by 1 mM IPTG, while chromosomal Bsp22^SPOT^ expression was attenuated with 50 mM MgSO_4_. Following 5 hours, cells were fixed and stained. Bsp22^SPOT^, magenta; Bsp22^ALFA^, green; bacterial cell surface, blue. The SIM images represent a single focal plane of cells and are representative of two independent experiments. Scale bars, 2 µm.

We next investigated the incorporation site of newly added Bsp22 subunits within the filament by employing an optimized tetracysteine tag (TC-tag) designed to bind biarsenical dyes (29). The SPOT-tag positioned between amino acids M138 and A139 of Bsp22 in *B. bronchiseptica* RB50 was substituted for the TC-tag, generating the *Bb bsp22*^TC^ strain. Importantly, this strain maintained T3SS-dependent cytotoxicity (Fig. 3A), confirming the continued functionality of the T3SS injectisome. To assess the addition of Bsp22 subunits to filaments, we performed a pulse-chase experiment using two different biarsenical dyes, resorufin arsenical hairpin (ReAsH) and fluorescein arsenical hairpin (FlAsH), with *Bb bsp22*^TC^ cells in solution. The bacteria were first labeled with ReAsH (pulse), washed, and then incubated with FlAsH (chase). As shown in a representative image of double-labeled cells (Fig. 3B), ReAsH predominantly stained the proximal segment of the filament, while FlAsH labeled the distal end of the growing filament. A 1-hour incubation with FlAsH was sufficient to clearly detect growing Bsp22 filaments. However, microscopic analysis also revealed substantial heterogeneity in filament staining and growth among individual *B. bronchiseptica* cells, as also shown in images from live monitoring using wide-field microscopy (Fig. S4A). Some cells showed no filaments, while others displayed growth only during the pulse (magenta) or chase (green) periods. Double-labeled filaments were also observed, likely due to incomplete saturation of ReAsH binding sites, allowing subsequent FlAsH labeling.

To further confirm the incorporation of Bsp22 monomers at the distal filament end, we next generated *Bb bsp22*^SPOT^ derivative strain carrying a plasmid that enables isopropyl β-d-1-thiogalactopyranoside (IPTG)-inducible expression of a Bsp22 variant, with ALFA-tag in between amino acid positions M138 and A139, *Bb bsp22*^SPOT^ // *bsp22*^ALFA^. After incubating this strain on coverslips for 3 hours, we added IPTG to induce Bsp22^ALFA^ expression and 50 mM MgSO_4_ to attenuate endogenous Bsp22^SPOT^ expression. After induction for 5 hours, cells were fixed and visualized by SIM microscopy (Fig. 3C). The data again revealed high heterogeneity in Bsp22 filament formation, including variability in both the presence and timing of Bsp22 monomers polymerization. Some cells did not respond to IPTG induction and displayed only Bsp22^SPOT^ filaments, while others began assembling Bsp22^ALFA^ filaments only after induction. Most filaments were chimeric, showing alternating incorporation of Bsp22^SPOT^ and Bsp22^ALFA^ subunits. Importantly, stronger anti-ALFA labeling was consistently observed at the distal filament end, indicating where newly synthesized Bsp22^ALFA^ was incorporated (see wide-field microscopy images in Fig. S4B).

In conclusion, these results demonstrate that Bsp22 filament formation on glass coverslips or in solution is an ongoing process. Furthermore, new Bsp22 subunits are incorporated at the distal end of the filament, resembling the assembly mechanism of flagellar filaments.

### T3SS injectisome functionality and BteA delivery do not require formation of long Bsp22 filaments

To further investigate Bsp22 filament formation and its physiological role, we analyzed these structures during infection of HeLa cells. Because the wild-type *B. bronchiseptica* RB50 strain produces a highly cytotoxic effector BteA, which rapidly kills HeLa cells (Fig. 1B), we generated a non-toxic derivative of *Bb bsp22*^SPOT^, *Bb bsp22*^SPOT^ / Δ*bteA*, by deleting the *bteA* coding region. We confirmed that this deletion did not interfere with the formation of Bsp22 filaments (Fig. S5A). Quantitative analysis of filament length and occurrence on the surface of *Bb bsp22*^SPOT^ / Δ*bteA* revealed similar characteristics to those of the parental strain. Specifically, filament length showed high variability, with the mean length increasing significantly between 0.5 and 3 hours of incubation on glass coverslip in *Bb*-SSM, confirming continuous growth (Fig. S5B and C).

**Fig 4:**
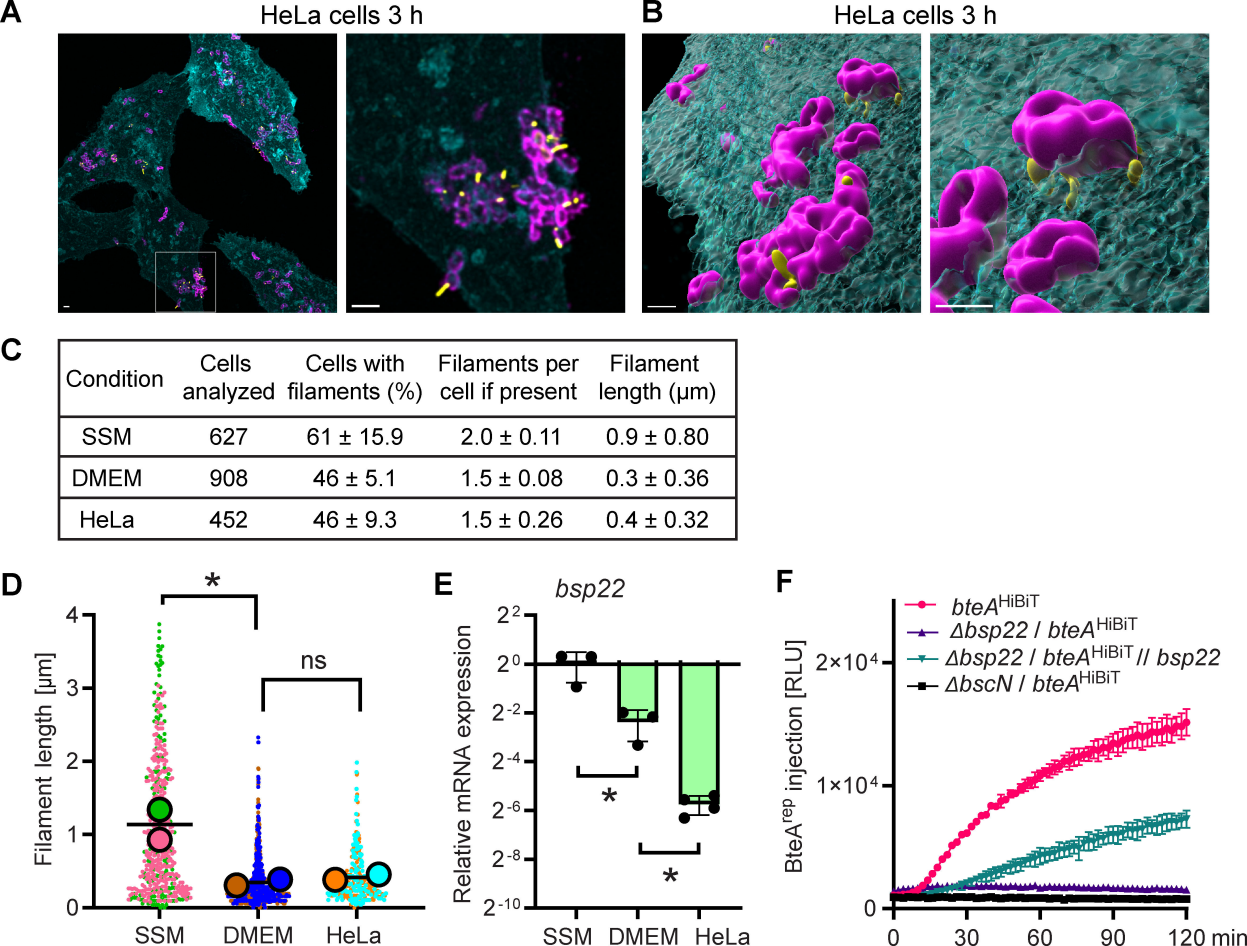
T3SS injectisome functionality and BteA delivery does not require formation of long Bsp22 filaments. (**A–B**) Visualization of Bsp22 filaments during HeLa cell infection. HeLa-PM-BFP were infected with *Bb bsp22*^SPOT^/ Δ*bteA* for 3 hours, fixed and stained. Bsp22, yellow; bacterial cell surface, magenta. The HeLa-PM-BFP cell surface (cyan) was detected through mTag-BFP2 fluorescence. (**A**) Maximal intensity (Z-max) projections of confocal Z-axis images. (**B**) 3D-surface rendering of all channels generated in Imaris. Images are representative of three independent experiments. Scale bars, 2 µm. (**C–D**) Quantitative analysis of Bsp22 filaments. (**C**) The percentage of *Bb bsp22*^SPOT^ / Δ*bteA* cells with filaments, filament count per cell, and filament length were quantified at 3 hours of bacteria cultivation on glass coverslip in *Bb*-SSM (SSM, Fig. S5), or DMEM-2%FBS (DMEM, Fig. S6), and at 3 hours post-infection of HeLa-PM-BFP (HeLa, Fig. 4A). Data are presented as mean ± SD, calculated from multiple microscopy fields in two independent experiments. (**D**) SuperPlots of filament lengths from two independent experiments. Distinct colors represent each experiment, with circles indicating the mean filament length of each experiment. The black bar shows the overall average. *P < 0.05, unpaired two-tailed *t*-test. (**E**) Downregulation of the *bsp22* gene expression. RNA was isolated at 3 hours of *Bb bsp22*^SPOT^ / Δ*bteA* cultivation in *Bb*-SSM (SSM), or DMEM-2%FBS (DMEM), and at 3 hours post-infection of HeLa-PM-BFP (HeLa). Relative *bsp22* mRNA levels were determined by reverse transcription-qPCR, with normalization to two housekeeping genes, *rpoB* and *dnaA*. Data represent mean ± SD from at least three biological replicates, each performed with three technical replicates. *P < 0.05, unpaired two-tailed *t*-test. (**F**) Assessment of BteA delivery. LgBit-expressing HeLa cells were infected with *Bb bteA*^HiBiT^ and its derivatives at an MOI of 5:1. Luminescence measurements were performed at 2-minute intervals and are reported as relative luminescence units (RLU). Shown are mean values ± SD of duplicate wells from a representative experiment out of two.

Using this strain, we infected HeLa cells stably expressing a plasma membrane-targeted blue fluorescent protein mTagBFP2, HeLa-PM-BFP, enabling us to track their surface. Three hours post-infection, samples were fixed and stained for Bsp22 and the bacterial cell surface. The Bsp22 filaments displayed diverse orientations, extending upward, away from, and toward the HeLa-PM-BFP cell surface. In several cases, Bsp22 filaments were oriented toward the HeLa cell membrane, closely interacting with the host surface (Fig. 4A and B). These interactions may represent physical bridges between the bacterium and the host cell membrane. Interestingly, Bsp22 filaments on bacteria in contact with HeLa cells appeared shorter than those observed on coverslips in *Bb*-SSM. However, when bacteria were cultivated on coverslips in Dulbecco’s Modified Eagle Medium (DMEM) with 2% fetal bovine serum (FBS), the medium used during HeLa cell infections, filament lengths were comparable to those observed during infection (Fig. S6). Quantitative analysis confirmed that although the number of filaments per bacterial cell remained unchanged, the mean filament length was significantly shorter in DMEM than in SSM, and no significant difference was observed between bacteria grown in DMEM for 3 hours and those infecting HeLa cells for 3 hours (Fig. 4C and D). Interestingly, the absence of filament growth correlated with a decrease in *bsp22* mRNA expression (Fig. 4E). This decrease was, however, more pronounced during infection of HeLa cells than in DMEM alone.

Importantly, despite their reduced length, these short Bsp22 filaments remained functional for BteA delivery into the HeLa cells. This was demonstrated using a split luciferase system (Fig. 4F), which relies on high-affinity complementation between a non-toxic BteA reporter (BteA^HiBiT^), in which the N-terminal 130-amino acid segment of BteA is fused to an 11-amino acid HiBiT tag, and the 18 kDa LgBiT fragment expressed in HeLa cells (23). Upon addition of the cell-permeable furimazine substrate, successful injection of the reporter is indicated by luminescence, thereby confirming BteA delivery into host cells. Deletion of the *bsp22* allele completely prevented BteA reporter delivery, whereas complementation with plasmid-encoded Bsp22 partially restored it, as shown in Fig. 4F. The incomplete restoration likely reflects differences in Bsp22 production from the plasmid compared to the native locus and/or the absence of coordinated co-expression with the cognate chaperone.

In summary, our data show that the growth of Bsp22 filaments is restricted during infection of HeLa cells, which is primarily due to the influence of the infection medium. This restriction is associated with reduced *bsp22* mRNA expression. However, the decrease in *bsp22* mRNA is more pronounced during interaction with HeLa cells than in DMEM medium alone, suggesting that host cell-derived signals also contribute to the regulation of *bsp22* mRNA independently of the infection medium. The resulting shorter filaments closely interact with the host cell surface, consistent with their role as a T3SS needle tip complex, and remain fully competent for mediating BteA delivery into host cells.

### *Bordetella* growth in cell cilia further downregulates the *bsp22* gene expression and tip filament formation

To investigate Bsp22 filament behavior in the context of airway ciliated epithelium, we employed an air-liquid interface (ALI) culture system of the human nasal epithelial cells (hNECs) grown on Transwell membranes (30–32). Primary nasal cells sampled from healthy donors were cultured under ALI conditions for four weeks to obtain well-differentiated hNECs, which were then infected with the non-cytotoxic *Bb bsp22*^SPOT^ / Δ*bteA* in DMEM supplemented with 2% FBS. The infection was carried out for 3, 6, and 24 hours, followed by fixation and staining of epithelial cells, bacteria, and Bsp22 filaments.

**Fig 5:**
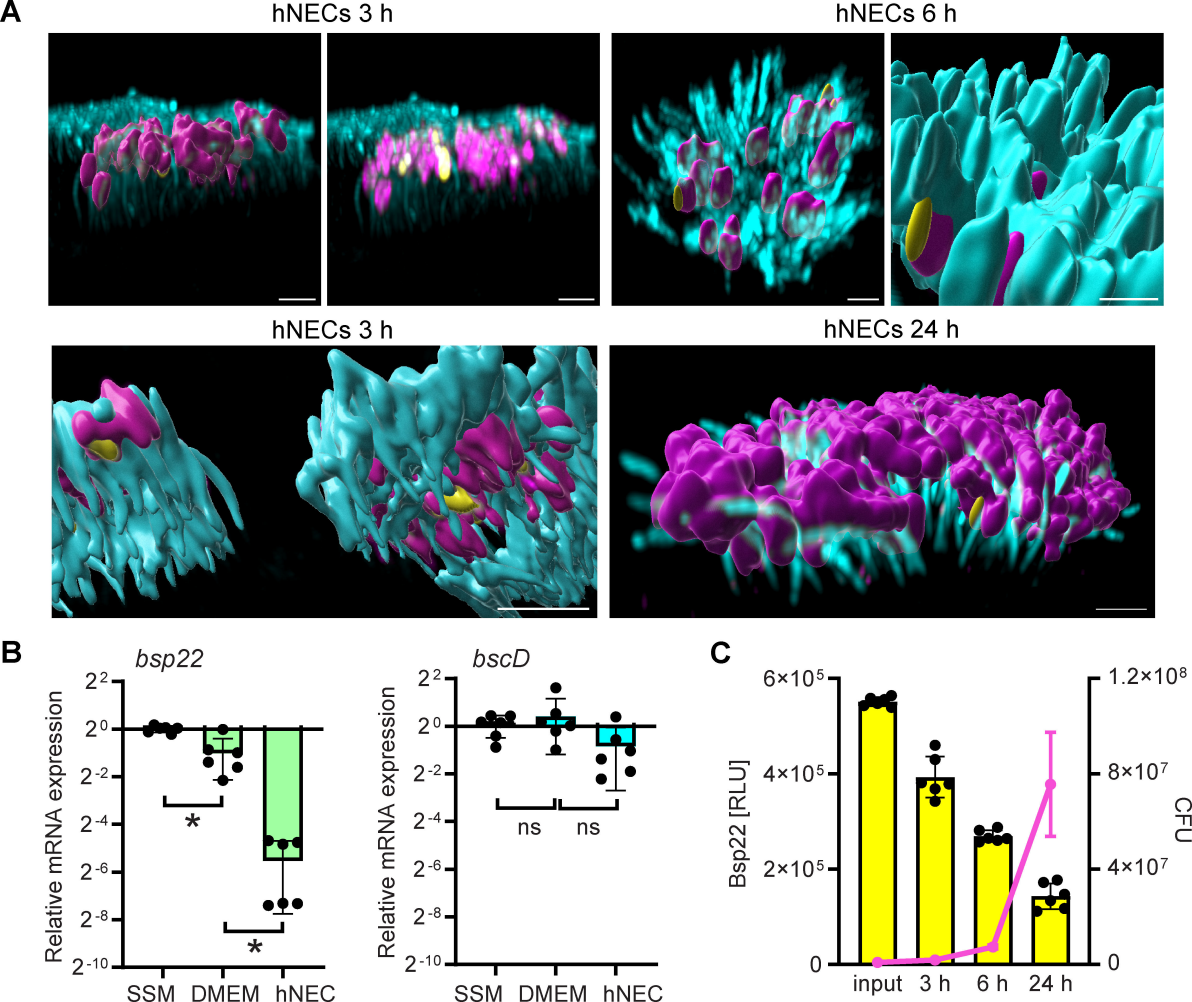
Bsp22 filaments become scarce during hNEC infection due to the downregulation of the *bsp22* expression. (**A**) Visualization of Bsp22 filaments during infection of hNECs with *Bb bsp22*^SPOT^/ Δ*bteA.* Cells were fixed at indicated time points and stained. Bsp22, yellow; bacterial cell surface, magenta; cilia, cyan. A 3D rendering of all channels was performed in Imaris. Images are representatives of three independent experiments. The bottom panel image on the left shows the bottom side of the cilia. Scale bars, 2 µm. (**B**) Downregulation of *bsp22* gene expression. RNA was isolated at 3 hours of *B. bronchiseptica* wild-type cultivation in *Bb*-SSM (SSM), or DMEM-2%FBS (DMEM), and at 3 hours post-infection of hNECs (hNEC). Relative mRNA levels of *bsp22* and *bscD* were determined by reverse transcription-qPCR, with normalization to two housekeeping genes, *rpoB* and *dnaA*. The data represent the mean values ± SD from six biological replicates, each performed with three technical replicates. *P < 0.05, unpaired two-tailed *t*-test. (**C**) Bsp22 protein levels are down-regulated during hNEC infection. hNECs were infected with *Bb bsp22*^HiBiT^/ Δ*bteA* for the indicated time. Bsp22 protein levels were quantified by luminescence measurements using the HiBiT system, and bacterial CFUs were determined by plating. The data represent the mean values ± SD from three biological replicates, each performed with two technical replicates.

Consistent with previous studies (33), and as shown in Fig. S7, staining of infected hNECs with wheat germ agglutinin (a lectin that binds cell carbohydrates), anti-ZO-1 (tight junction marker), and anti-acetylated tubulin (cilia marker) antibodies revealed that *B. bronchiseptica* was specifically localized to the host cell cilia. As further illustrated in Fig. 5; Fig. S8, at 3 hours post-infection, *Bb bsp22*^SPOT^ / Δ*bteA* cells were attached to the tips of the cilia. Over the course of the experiment, bacteria multiplied and grew within the cilia in clusters, gradually advancing to the base of the cilia. By 24 hours post-infection, in most of the ciliated cells, the space between the cilia was densely colonized by bacteria. However, the ciliated cells shown in Fig. S8 appears to contain relatively few bacteria at 24 hours post-infection, likely due to differences in the imaging depth and the variation in bacterial density on the surface of the Transwell membrane.

Bsp22 filaments were scarce throughout the hNEC infection. The few filaments that were detected appeared to align parallel to the *B. bronchiseptica* cells and the host cell cilia (Fig. 5A; Fig. S8). However, due to the complexity of the samples and the low number of filaments, quantification of this alignment was not possible. Consistent with HeLa cell infection, *bsp22* mRNA levels declined significantly by 3 hours post-infection, with a greater reduction in hNEC infection compared to the infection DMEM medium alone (Fig. 5B). Importantly, this downregulation was specific to *bsp22* mRNA, as the mRNA levels of *bscD*, encoding the inner membrane ring component of the injectisome, remained unchanged (Fig. 5B). To confirm the decline in Bsp22 filament formation, we assessed Bsp22 protein using a reporter strain *Bb bsp22*^HiBiT^ / Δ*bteA*, carrying a HiBiT-tag inserted between amino acids M138 and A139 of Bsp22. This allowed luciferase-based quantification through complementation with the 18 kDa LgBiT fragment (34, 35). Luciferase activity measurements at different time points post-infection revealed a decline in Bsp22 levels despite bacterial proliferation (Fig. 5C). This confirms that Bsp22 production decreases over time during infection.

To further validate Bsp22 downregulation during the conditions mimicking natural infection, we employed an alternative infection protocol in which *B. bronchiseptica* was added directly to hNECs cultured at ALI in five 1-µL drops of SSM and visualized at 3 and 24 hours post-infection. As shown in Fig. S9, this infection protocol resulted in more abundant detection of Bsp22 filaments at 3 hours post-infection, consistent with the observation that DMEM supplemented with 2% FBS suppresses filament growth. However, by 24 hours, Bsp22 filaments were no longer detectable, highlighting the contribution of the host ciliated epithelium to the suppression of Bsp22 filament formation.

In summary, during hNEC infection, *B. bronchiseptica* specifically colonized the cilia where it multiplied and formed bacterial cell clusters. Bsp22 filaments were sparse and appeared to align with the host cilia. The reduced filament formation correlated with a decrease in the *bsp22* mRNA expression, independent of mRNA for *bscD*. Furthermore, reporter assays with *Bb bsp22*^HiBiT^ / Δ*bteA* confirmed that Bsp22 protein levels decreased over time. The results of the alternative hNEC infection scheme, which was designed to better mimic natural infection and avoided infection using DMEM supplemented with 2% FBS, also demonstrated downregulation of Bsp22 filaments during infection. Taken together, these results suggest that *B. bronchiseptica* tightly regulates the formation of the T3SS tip filament in response to the environment and interaction with the host cell, with reduced Bsp22 synthesis likely playing a key role in this adaptation.

## DISCUSSION

In this study, we provide novel insights into the assembly and dynamics of the T3SS-tip filament formed by the Bsp22 protein in *B. bronchiseptica* and *B. pertussis*. By engineering a short peptide tag into a surface-exposed loop of Bsp22, we were able to use super-resolution imaging to dissect the process of filament formation and demonstrate its regulation under different growth conditions. Considering the distinct categorization of Bsp22 within the T3SS tip complex families (21), such investigation is crucial for understanding the assembly and function of diverse T3SS injectisome families and Bsp22 function in classical *Bordetella* species.

The T3SS injectisome of *B. bronchiseptica* differs from the T3SS injectisomes of other gram-negative bacteria, such as *Salmonella* and *Shigella*, by its elongated extracellular filament. This characteristic is reminiscent of T3SS injectisomes found in phytopathogens, which, unlike the typical short needle filaments, produce flexible pilus-like extensions known as the Hrp pilus (36, 37). Additionally, T3SS injectisomes of A/E pathogens, comprising EPEC (enteropathogenic *E. coli*), EHEC (enterohemorrhagic *E. coli*), and the murine pathogen *Citrobacter rodentium*, also form extended structures known as EspA filaments (17, 38, 39). These filaments connect the needle filament to the translocon pore in the host cell membrane and are formed through the polymerization of the tip protein EspA (17, 19, 20, 40, 41).

Despite the minimal sequence similarity between Bsp22 and EspA and the definition of Bsp22 as the founding member of a novel T3SS-tip complex family (21), the monomeric structure of Bsp22 predicted by AlphaFold resembles that of monomeric EspA (19, 20, 42). Both proteins possess a coiled-coil structure between their N- and C-terminal α-helices, separated by an extended, ordered loop. In EspA, this loop accommodates hypervariable amino acids responsible for antigenic polymorphisms and allows for amino acid insertions (26, 27). Similarly, our study demonstrated that the corresponding loop in Bsp22 is permissive, which allowed for peptide display and facilitated the detailed super-resolution imaging of Bsp22 filaments using commercially available nanobodies.

We observed that both *B. bronchiseptica* and *B. pertussis* can produce long and flexible Bsp22 filaments that extend several µm from bacterial cells on glass coverslips. Although the average number of filaments per cell is similar for *B. bronchiseptica* and *B. pertussis*, filament elongation might be slower in *B. pertussis*. This difference could be due to species-specific variations in metabolism or regulation of T3SS activity. The denser filament network observed for *B. bronchiseptica* on glass coverslips is also likely due to a higher number of attached cells compared to *B. pertussis*, possibly resulting from the faster growth rate and/or better attachment of *B. bronchiseptica*. Quantitative analysis showed that approximately 50%–80% of bacteria have filaments, with an average of two filaments per bacterium. This is significantly less than the number of EspA filaments in EPEC (approximately 12 per bacterium) (43), or the injectisome counts in *Salmonella* (10–100 per bacterium) (12) or *Yersinia* (30–100 per bacterium) (14). Unlike needle filaments, which have a defined length control mechanism (44, 45), the Bsp22 filaments grow continuously on a glass coverslip in *Bordetella* SSM cultivation medium. This ongoing growth, characterized by high variability in filament length, suggests that the Bsp22 filament is a dynamic structure. Furthermore, we demonstrated that newly secreted Bsp22 subunits are added at the distal end of the filament, a process similar to assembly mechanisms seen in flagellar (46) and EspA (47) filaments but different from the basal growth pattern observed in type I and type IV pili (48).

The Bsp22 filament growth is regulated by environmental conditions. When bacteria are incubated in the SSM, Bsp22 filaments grow continuously and reach lengths over two μm. However, when bacteria are exposed to infection conditions, either when grown in DMEM supplemented with 2% FBS or during HeLa cell infection, filaments become significantly shorter, although the number of filaments per cell remains constant. A key molecular correlate to this phenotypic change in *B. bronchiseptica* is the significant decrease in *bsp22* mRNA expression. Importantly, the reduction in *bsp22* transcript levels is more pronounced during actual cell infection than in the DMEM alone. This suggests that, in addition to the composition of the infection medium or the absence of stimulatory signals present in the SSM, host cell contact further downregulates *bsp22* mRNA expression. Importantly, we also observed filament downregulation during hNEC infection when bacteria were applied more naturally, in 1-μl drops of SSM directly onto the cell surface. Indeed, the growth of Bsp22 filaments may be influenced by the intracellular levels of *bsp22* mRNA and availability of Bsp22 protein subunits, similar to the regulation proposed for EspA filaments (47). However, the mechanisms behind *bsp22* mRNA downregulation remain unclear, as does the potential involvement of additional posttranscriptional or posttranslational processes in controlling Bsp22 protein production and/or secretion.

T3SS biogenesis in gram-negative bacteria is a tightly regulated process in which substrates are secreted in a specific order, which involves at least two substrate specificity switches (49). In *B. pertussis*, the secreted protein BP2259, a homolog of *Yersinia* YscX, is required for activating Bsp22 secretion (50). This mirrors the role of YscX in *Yersinia*, which is required for the export of the first substrates (51). Additionally, we recently determined the crystal structure of the *Bordetella* BopN protein and found that it closely resembles gatekeeper proteins that regulate access to the T3SS channel from the bacterial cytoplasm. However, while BopN controls the secretion of the BteA effector, it has little to no impact on Bsp22 secretion (23). Proteins involved in regulating Bsp22 filament growth are likely encoded within the *btr* (*Bordetella* type III regulation) locus, which is located next to the *bsc* locus that encodes the T3SS injectisome components (52). These include the BtrS-BtrA/BspR protein regulatory system and the partner-switching proteins BtrU, BtrW, and BtrV, which act at both transcriptional and translational levels to control injectisome components and substrate export (52–55). However, the molecular mechanism underlying the downregulation of *bsp22* mRNA during host cell contact and/or environmental sensing remains unclear.

Interestingly, the increased intracellular c-di-GMP levels also repress *bsp22* transcription (56), suggesting that activation of a specific diguanylate cyclase upon host contact and/or sensing a small chemical signal via a sensory domain could be involved. Another intriguing possibility is that host cell contact prevents further polymerization of Bsp22 filaments, leading to accumulation of Bsp22 in the bacterial cytosol, which in turn could trigger a negative feedback mechanism that represses transcription of *bsp22*. Alternatively, direct docking of the T3SS injectisome to the host plasma membrane may trigger a feedback mechanism, through mechanical or ion sensing via the T3SS conduit, analogous to the translocation “switch” mechanisms described in other T3SS systems (57). In the future, it will be important to dissect these multiple regulatory layers and connect them to molecular signals that control Bsp22 expression and filament assembly.

Intriguingly, previous studies have reported upregulation of *bsp22* mRNA and/or increased Bsp22 secretion in *B. pertussis* upon host cell contact and in both *B. pertussis* and *B. bronchiseptica* following exposure to blood and/or serum (50, 58, 59). However, these results were obtained under conditions different from those used in our study. Specifically, Bibova et al. (58) analyzed *B. pertussis* Tohama I recovered from RAW 264.7 macrophages and then transferred into SS medium instead of monitoring the bacteria during direct interaction with the host cells. It is plausible that the long-passaged, laboratory-adapted Tohama I strain reactivated its virulence program during infection, which resulted in increased Bsp22 expression (60). Similarly, Gestal et al. (59) found that cultivation in 100% sheep serum increased Bsp22 expression compared to SS medium, which suggests enhanced Bsp22 expression under host-associated or pre-contact conditions. Although filament formation was not visualized in these studies, they confirm that Bsp22 levels are responsive to specific environmental cues. In the future, it will be important to analyze whether these signals also promote filament formation.

In our experiments, *bsp22* mRNA levels were consistently downregulated during infection of both HeLa cells and hNECs. Moreover, the downregulation of the *bsp22* transcript in hNECs was in contrast to the stable mRNA expression of another T3SS component, the inner membrane ring protein, BscD. This suggests that the regulation was specific to Bsp22 and not a general repression of T3SS. Such targeted regulation may allow *Bordetella* to adapt its T3SS injectisome in response to environmental cues, potentially preventing excessive filament formation after attachment. Indeed, downregulation of the *bsp22* transcript correlated with reduced Bsp22 protein levels and fewer detectable filaments during infection with *B. bronchiseptica*. This decrease was more pronounced in hNECs at 3 hours post-infection than in HeLa cells when the bacteria were applied in DMEM, which could be due to filament instability caused by ciliary beating and/or more efficient protease-mediated degradation. Another, although less likely, possibility is that the complexity of the hNEC samples limited the visualization of the filaments.

The exact reason why *Bordetella* species and A/E pathogens form filamentous tips on their injectisomes is unclear. In A/E pathogens, it has been suggested that EspA filaments play a role in the early stages of infection, possibly facilitating the traversal of the mucus barrier and cell attachment of A/E pathogens. Indeed, these filaments are not maintained throughout infection and are absent in mature A/E lesions once intimate attachment is established (17, 61). A similar function could be hypothesized for *Bordetella* species, where Bsp22 filaments may promote an initial interaction with host cells. Nevertheless, the function of Bsp22 filaments beyond their role in T3SS-mediated BteA delivery remains to be elucidated.

Although *B. bronchiseptica* can persist in environmental reservoirs, a role of Bsp22 filaments in environmental persistence seems unlikely, as T3SS expression, including Bsp22, is controlled by the BvgAS two-component system, which is active in the Bvg^+^ (virulent) phase during host colonization (54, 62, 63). This regulatory pattern argues against a role of Bsp22 filaments outside the host. It could be hypothesized that Bsp22 filaments contribute to bacterial aggregation in airway mucus, facilitate early attachment to host epithelial surfaces and/or help *Bordetella* cells position their injectisomes for efficient effector translocation within the complex architecture of ciliated epithelia. Importantly, our data show that contact with host cells does not stimulate filament elongation, suggesting that Bsp22 filaments function in the pre-contact and/or early contact phase, where they may help to stabilize interactions with the host surface. Once stable attachment is established, these structures may become unnecessary or even counterproductive, potentially impairing intimate contact. Downregulation of their length upon contact with the host cell could promote a tighter connection between the T3SS needle and the host membrane, which might enable more efficient BteA translocation, while also reducing exposure of the immunogenic Bsp22 protein to the host immune system (21).

In conclusion, our study provides new insights into the assembly and dynamics of Bsp22 filaments in classical *Bordetella* species, both in an artificial system using glass coverslips and during host cell infection. This was achieved through fluorescence imaging enabled by the incorporation of short peptide tags into Bsp22 without compromising injectisome functionality. Our findings demonstrate that Bsp22 filaments are dynamic structures whose growth is tightly controlled by bacterial regulatory mechanisms and environmental factors. These results highlight the adaptability of the *Bordetella* T3SS.

## MATERIALS AND METHODS

### Experimental model details

#### Cell lines

HeLa cells (ATCC, Cat# CCL-2, human cervical adenocarcinoma), HeLa-LgBit (HeLa cells constitutively expressing LgBit [23]), and HeLa-PM-BFP (HeLa cells constitutively expressing plasma membrane-targeted blue fluorescent protein, mTagBFP2; see below) were cultured in DMEM with 10% heat-inactivated FBS (DMEM-PhenolRed-10%FBS) at 37°C and 5% CO_2_. For microscopy purposes, DMEM without a phenol-red indicator, supplemented with either 10% or 2% heat-inactivated FBS (DMEM-10%FBS and DMEM-2%FBS, respectively), to minimize cell autofluorescence was used.

HeLa-PM-BFP stable cell line was generated by lentiviral transduction of the parental HeLa cell line. The pLJM1-PM-BFP vector used for virus production was prepared by subcloning the plasma membrane targeting sequence, Rpre (64), in frame with mTagBFP2 coding sequence at its C-terminus. Vesicular stomatitis virus (VSV)-pseudotyped viruses were then produced by co-transfecting 6 µg of pLJM1-PM-BFP, 6 µg of pCMV-VSV-G, and 6 µg of psPAX2 plasmids into 293T cells grown in a 10 cm dish using Lipofectamine 2000 (Invitrogen). The cell culture supernatant was collected 48 h after transfection and used to transduce parental HeLa cells in the presence of polybrene (8 µg/mL). Twenty-four hours after transduction, cells were split, selected by puromycin (0.5 µg/mL), and sorted by flow cytometry to obtain single-cell clones of HeLa-PM-BFP cells.

The feeder cell line for nasal epithelial cells, 3T3-J2 (Kerafast, Cat# EF3003, embryonic mouse fibroblasts), was cultured in DMEM with 10% bovine calf serum and antibiotics (0.1 mg/mL streptomycin and 1,000 U/mL penicillin). To arrest cell proliferation of feeder 3T3-J2 cells, mitomycin C was added to the medium at a final concentration of 4 µg/mL, and cells were incubated for 2 hours at 37°C and 5% CO_2_.

#### ALI cultures of hNECs

hNECs were collected from the anterior nares of healthy donors using a cytology brush. After digestion with TrypLE Select (Gibco, Cat# 12604013) for 5 minutes at 37°C, the resulting single cells were centrifuged (5 minutes; 350 *g*) and gently resuspended in the NEC medium, specified in Table S1. To allow for their conditional reprogramming, cells were seeded into a T25 flask on mitomycin-treated 3T3-J2 fibroblasts in the NEC medium, as previously reported (31, 32). Once the hNEC population had sufficiently expanded, 5 × 10^4^ cells were plated onto the apical side of a collagen-coated 6.5 mm Transwell filter (Corning Costar, Cat# 3470) in 200 µL of apical and 600 µL of basolateral NEC medium without fungin. After 72 hours, during which the cells reached confluency, the apical medium was removed (air-lifting), and the basolateral medium was replaced by the differentiation ALI medium (Table S1). The ALI medium was replaced three times per week. The air-lifting defined day 0 of the ALI culture, and the experiments were conducted between days 25 to 28. To prevent mucus accumulation on the apical side, the cells underwent a 30-minute apical wash with phosphate-buffered saline (PBS) every 5–7 days starting from day 14 of the ALI culture. The differentiation and integrity of ALI cultures were verified through visual inspection and measurement of transepithelial electrical resistance before each experiment. Additionally, on day 23 of the ALI culture, the basolateral ALI medium was replaced with the ALI medium without antibiotics, with a minimum of two medium changes occurring prior to the experiment.

#### Bacterial strains

The bacterial strains used in this study are detailed in Table S2. Plasmid construction was carried out using *E. coli* strain XL1-Blue, while plasmid transfer into *B. bronchiseptica* RB50 or *B. pertussis* B1917 was performed by bacterial conjugation using *E. coli* strain SM10λpir. *E. coli* strains were cultivated at 37°C on LB agar or in LB broth, supplemented with 100 µg/mL of ampicillin. Wild-type *B. bronchiseptica* RB50 and *B. pertussis* B1917, along with their derivatives, were cultivated on Bordet-Gengou (BG) agar medium (Difco) supplemented with 1% glycerol and 15% defibrinated sheep blood (LabMediaServis) at 37°C and 5% CO_2_ for 48 hours (*B. bronchiseptica*) or 72 hours (*B. pertussis*). For the growth of liquid *B. bronchiseptica* and *B. pertussis* cultures at 37°C, modified SSM, *Bb*-SSM, and *Bp*-SSM, respectively, with reduced concentrations of L-glutamate (monosodium salt) and no FeSO_4_.7H_2_O added, as specified in Table S1, were used. Bacteria in the exponential growth phase, with OD_600_ ~1.5 for *B. bronchiseptica* and OD_600_ ~1.0 for *B. pertussis*, were used for the experiments. Culture medium of *B. bronchiseptica* RB50 harboring pBBRI plasmid was further supplemented with chloramphenicol (30 µg/mL).

### Method details

#### Mutagenesis and plasmid construction

Plasmids used in this study are listed in Table S3. Plasmids were constructed using the Gibson assembly strategy (65). PCR amplifications were performed using Herculase II Phusion DNA polymerase (Agilent, Cat# 600675) from the chromosomal DNA of *B. bronchiseptica* RB50 or *B. pertussis* B1917 as the template. The coding sequence of mTagBFP2 was amplified from mTagBFP2-pBAD (Addgene [66]), whereas lacIq-Ptac-inducible promoter was amplified from pUC18T-miniTn7T-gm-lacIq-Ptac (Addgene [67]). All constructs were verified by DNA sequencing (Eurofins Genomics). The pBBRI plasmid encoding lacIq-Ptac-*bsp22*^ALFA^ and Pbsp22-*bsp22* was introduced into *B. bronchiseptica* by conjugation, as described previously (68). Mutant *B. bronchiseptica* and *B. pertussis* strains were constructed by homologous recombination using the suicide allelic exchange vector pSS4245, following established protocols (68). The presence of the introduced mutations was confirmed by PCR amplification of the relevant regions of the *Bordetella* chromosome, followed by agarose gel analysis and DNA sequencing (Eurofins Genomics).

#### Determination of cellular cytotoxicity

HeLa cells were seeded at a density of 5 × 10^4^ per well in a 96-well plate in DMEM-2%FBS. The next day, HeLa cells were infected with exponential *B. bronchiseptica* culture at the indicated MOI. After centrifugation (5 minutes; 300 *g*) to increase infection efficiency, HeLa cells were incubated for 3 hours at 37°C and 5% CO_2_. Cellular cytotoxicity was measured as lactate dehydrogenase (LDH) release into the cell culture medium using the CytoTox 96 assay (Promega, Cat# G1780), according to the manufacturer’s instructions.

#### Preparation of glass coverslips for fluorescence imaging and antibodies used

High-precision coverslips (#1.5 H, 18 × 18 mm, Marienfeld, Cat# 0107032) were immersed in 1 M HCl for 1 hour, followed by five rinses in deionized water (dH_2_O) and air-drying. Subsequently, the coverslips were stored in 96% ethanol. Unless stated otherwise, uncoated coverslips were used. For SIM visualization of pulse-chase labeling of Bsp22^TC^, coverslips were coated with 0.01% poly-L-lysine (Boster Biological Technology, Cat# AR0003) for 15 minutes, washed twice with dH_2_O, and air-dried. Antibodies and other labels used in this study, including their working dilutions, are listed in Table S4.

#### Visualization of Bsp22^SPOT^ on glass coverslips

To balance bacterial growth on coverslips, different colony-forming units (CFUs) of *B. bronchiseptica* or *B. pertussis* were distributed in the *Bb*-SSM and *Bp*-SSM, respectively, on each uncoated high-precision glass coverslip within a 6-well plate. The CFU amounts used were 1.5 × 10^9^ CFU for a 0.5-hour incubation, 6 × 10^8^ CFU for a 1- and 2-hour incubation, 1.5 × 10^8^ CFU for a 3-hour incubation, and 3 × 10^7^ CFU for a 6-hour incubation. After dilution and distribution, the bacteria were centrifuged (3 minutes; 300 *g*) to facilitate bacterial adhesion. The centrifugation step was necessary because uncoated glass coverslips (without poly-L-lysine or other adhesives) were used to avoid possible interference with Bsp22 filament formation. Without this initial centrifugation, no or very little bacterial attachment was observed. The plates were then incubated at 37°C and 5% CO_2_. At the end of each incubation, coverslips were rinsed with PBS, and adherent cells were fixed with 4% paraformaldehyde (PFA) for 20 minutes at room temperature (RT). Coverslips were washed with PBS (3 × 5 minutes) and blocked with 4% bovine serum albumin (BSA) in PBS (4%BSA-PBS) for 1 hour at RT. The SPOT-tag nanobody conjugated with ATTO488 (Spot-label ATTO488) at a dilution of 1:4,000 and a rabbit anti*-Bordetella* serum (provided by Dr. Vecerek, Institute of Microbiology, Prague, Czech Republic) at a dilution of 1:1,000 in 1% BSA in PBS (1%BSA-PBS) were applied overnight at 4°C. The following day, coverslips were washed in PBS (3 × 5 minutes) and incubated with anti-rabbit IgG-DyLight 405 conjugate at a dilution of 1:500 in 1% BSA-PBS for 1 hour at RT. After washing in PBS (3 × 5 minutes) and brief rinses in dH_2_O (10×), coverslips were mounted on glass slides using the Vectashield (Vector Laboratories, Cat# H-1000) mounting medium and SecureSeal Imaging Spacers (Grace Bio-Labs, Cat# 654002).

#### Pulse-chase labeling of Bsp22^TC^

Pulse-chase labeling of *Bb bsp22*^TC^ with the biarsenic reagents ReAsH (ReAsH-EDT_2_, Cayman Chemical, Cat# 19767) and FlAsH (FlAsH-EDT_2_, Cayman Chemical, Cat# 20704) was carried out in reducing *Bordetella* labeling medium (BLM) containing 0.5 mM tris(2-carboxyethyl)phosphine hydrochloride. BLM, developed in this study, is based on M9 minimal and SSM media, and its composition is detailed in Table S1. *Bb bsp22*^TC^ cells (1.5 × 10^9^ CFU) were pelleted (10 minutes; 1,200 *g*), resuspended in BLM, and incubated for 30 minutes at 37°C to allow reduction of extracellular cysteines. ReAsH was then added at a final concentration of 1 µM, and the bacteria were incubated stationary in an Eppendorf tube at 37°C. The progress of labeling was checked by wide-field microscopy when 5 µL samples of the culture were spotted onto a coverslip and covered with an agarose pad prepared in PBS. The coverslip was attached to a support and observed under a wide-field microscope (Olympus). When Bsp22 filaments were well-discernible on the cells (after 220 minutes), ReAsH-EDT_2_ was removed by centrifugation (10 minutes; 1,200 *g*). Pelleted bacteria were resuspended in BLM and incubated for 30 minutes at 37°C. FlAsH-EDT_2_ was then added at a final concentration of 1 µM, followed by incubation at 37°C. The progress of labeling was again checked by wide-field microscopy of culture spotted onto a coverslip and covered with an agarose pad. When double-labeled Bsp22 filaments were visible (after 60 minutes), the bacteria were applied onto poly-L-lysine-coated coverslips and allowed to adhere for 10 minutes. After rinsing with PBS once, the cells were fixed with 4% PFA for 20 minutes at RT. The coverslips were washed with PBS (3 × 5 minutes), rinsed in dH_2_O (10×) and mounted using Vectashield onto glass slides.

#### Induction and labeling of Bsp22^ALFA^

For Bsp22^ALFA^ expression, *Bb bsp22*^SPOT^ bacteria harboring the plasmid pBBRI-*lacIq*-Ptac-*bsp22^ALFA^* (Table S2) were seeded on coverslips and centrifuged (3 minutes; 300 *g*) to facilitate adhesion. After 3 hours of incubation at 37°C and 5% CO_2,_ the expression of chromosomal *bsp22*^SPOT^, regulated by the *Bordetella* BvgAS system, was attenuated by adding MgSO_4_ to a final concentration of 50 mM. Simultaneously, expression of *bsp22*^ALFA^ was induced by 1 mM IPTG, and bacteria were incubated for an additional 5 hours at the same conditions. Post-incubation, the coverslips were rinsed with PBS, and the adherent cells were fixed with 4% PFA for 20 minutes at RT. Following fixation, coverslips were washed with PBS (3 × 5 minutes), blocked with 4% BSA-PBS for 1 hour at RT, and incubated overnight at 4°C with a combination of ALFA nanobody conjugated to ATTO488 at a dilution of 1:500, Spot-label ATTO594 at a dilution of 1:4,000, and the rabbit anti-*Bordetella* serum at a dilution of 1:500 in 1%BSA-PBS. The following day, coverslips were washed in PBS (3 × 5 minutes) and incubated for 1 hour at RT with anti-rabbit IgG-DyLight 405 conjugate at a dilution of 1:500 in 1% BSA-PBS. After washing with PBS (3 × 5 minutes) and rinsing in dH_2_O (10×), the coverslips were mounted on glass slides using the Vectashield mounting medium.

#### Visualization of Bsp22^SPOT^ during HeLa cell infection

The HeLa-PM-BFP cells were seeded at a density of 2.5 × 10^5^ in a 12-well plate using DMEM-10%FBS and allowed to grow overnight. The following day, the medium was replaced with DMEM-2%FBS, and the cells were infected with *Bb bsp22*^SPOT^ / Δ*bteA* at MOI 50:1 followed by centrifugation (3 minutes; 300 *g*) to enhance the infection efficiency. Three hours post-infection at 37°C with 5% CO_2_, the cells were rinsed with PBS and fixed with 4% PFA for 20 minutes at RT. Coverslips were washed with PBS (3 × 5 minutes), blocked with 4% BSA-PBS for 1 hour at RT, and incubated with Spot-label ATTO488 at a dilution of 1:5,000 and the rabbit anti-*Bordetella* serum at a dilution of 1:1,000 in 1%BSA-PBS overnight at 4°C. Subsequently, the coverslips were washed in PBS (3 × 5 minutes) and incubated with anti-rabbit IgG-AF647 conjugate at a dilution of 1:600 in 1% BSA-PBS for 1 hour at RT. Finally, following washing in PBS (3 × 5 minutes) and rinsing in dH_2_O (10×), coverslips were mounted on glass slides using the Vectashield mounting medium.

#### Visualization of Bsp22^SPOT^ in ALI cultures of hNECs

Differentiated ALI cultures of hNECs were apically infected with *Bb bsp22*^SPOT^ / Δ*bteA* using 100 µL of DMEM-2%FBS at 37°C with 5% CO_2_. For 3- and 6-hour infections, 5 × 10^6^ CFUs were applied per a Transwell membrane, and for the 24-hour infection, 10^5^ CFUs were used. After 3 hours, the medium was carefully removed, and the cultures were either processed immediately or further incubated under ALI conditions without washing. At indicated time points, membranes were rinsed with PBS and fixed with 4% PFA for 20 minutes at 37°C. Following fixation, membranes were washed with PBS (3 × 5 minutes), and samples were permeabilized with 0.2% Triton-X100 (TX-100) in PBS for 10 minutes at RT. Subsequently, membranes were washed with PBS supplemented with 0.05% TX-100 (PBST) and blocked with 4% BSA in PBST for 1 hour at RT. Spot-label ATTO594 at a dilution of 1:5,000, rabbit anti-*Bordetella* serum at a dilution of 1:500, and mouse monoclonal anti-acetylated tubulin antibody at a dilution of 1:500 in 1%BSA-PBS were applied overnight at 4°C. The following day, the membranes were washed with PBST (3 × 5 minutes) and incubated with the anti-rabbit IgG-DyLight 405 conjugate at a dilution of 1:500 and the anti-mouse IgG-AF488 conjugate at a dilution of 1:500 in 1% BSA in PBST for 1 hour at RT. This was followed by washes with PBST (3 × 5 minutes) and a brief rinse with dH_2_O. Finally, the membranes were dissected from Transwells, placed inside SecureSeal Imaging Spacers attached to the glass slides, covered with the Vectashield mounting medium, and sealed with high-precision coverslips.

#### Microscopy

Wide-field microscopy was employed to check the progress of pulse-chase labeling of Bsp22^TC^ and to inspect samples obtained with the above-described methods prior to examining them by SIM or confocal microscopy. The Olympus IX83 inverted microscope equipped with the UPLXAPO100XOPH NA 1.45 objective and sCMOS camera Prime 95B (Photometrics, USA) with high quantum efficiency, large field of view, and large pixel size of 11 µm was used. mTagBFP-2 and DyLight405 were observed using the excitation filter 402/15 nm, the emission filter 455/50 nm, and a quadruple dichroic mirror DAPI/FITC/TRITC/CY5. ATTO488 and AF488 were excited through the filter 490/20 nm and captured by the 525/36 nm emission filter. When using ATTO594 and AF568, the excitation filter 572/35 nm and the emission filter 632/60 nm were combined with a triple dichroic mirror DAPI/FITC/TxRed.

Confocal images were acquired with Leica STELLARIS 8 FALCON equipped with wide-range (440–790 nm), light laser with pulse picker (WLL PP), and highly sensitive hybrid detectors operated by the LAS X software. The objective HC PL APO 63×/1.40 OIL CS2; FWD 0.14 MM was used with the type F immersion oil (Leica, 11513859). mTagBFP2 and DyLight405 were excited with the 405 nm cw diode laser and other dyes with the WLL-PLL laser with the following settings: AF488 (491 nm), ATTO488 (501 nm), ATTO594 (602 nm), and AF647 (644 nm). Z-stacks were acquired with steps of 0.15 µm, and pixel size was set to meet Nyquist sampling criteria for deconvolution with the Huygens software (SVI, Netherlands).

The DeltaVision OMX imaging platform was used to acquire images with 3D structured illumination. The system was equipped with the PLAN APO N 60× oil objective, NA 1.42; FWD 0.15; CG 0, four PCO, and an Edge 5.5 sCMOS camera (readout speeds 95 MHz, 286 MHz, 15-bit, pixel size 6.5 µm). To excite fluorescent proteins and the labels on the antibodies, 405 nm diode and 488 nm and 568 nm OPSL were used in combination with emission filters 435.5/31, 528/48 and 609/37 nm. SoftWoRx software (Applied Precision, USA) was used for image reconstruction and registration.

#### Image processing

Acquired confocal images were deconvolved using Huygens Professional 22.10 software (SVI, https://svi.nl). Orthogonal views, 3D, and surface rendering were prepared in Imaris 10.0, the image analysis software (Bitplane/Oxford Instruments, https://imaris.oxinst.com/). Further image processing, consisting of cropping and brightness/contrast adjustment, was performed in FIJI (28), NIH Bethesda, https://imagej.nih.gov). Final images were assembled in Adobe Illustrator (Adobe).

#### Quantitative analysis of Bsp22 filaments

Microscopic images were analyzed by a custom-made macro for Fiji (28). Briefly, bacteria are segmented using Cellpose (69) integrated into Fiji via BIOP/ijl-utilities-wrappers. Bsp22 filaments are then manually traced one cell at a time, allowing for accurate measurement of overlapping filaments. Automatically generated traces are provided as suggestions. The organization of the results makes every measurement verifiable in the original image. The macro uses MorphoLibJ (70), BioVoxxel (71), and BIOP plugins. A detailed explanation of the function of the macro and its operation is included in the macro code. The code is available on https://github.com/LMCF-IMG/T3SS-filaments. SuperPlots have been used in Fig. 2C and 4D; Fig. S6 (72) to communicate experimental reproducibility in measured lengths of the filaments. Versions of plugins working with the macro (most notably ijl-utilities-wrappers-0.9.7) and full analysis output allowing for independent review of complete individual measurements are available upon request.

#### Reverse transcription-qPCR analysis of gene expression in *B. bronchiseptica*

To analyze gene expression in *B. bronchiseptica* following infection of HeLa-PM-BFP and hNECs and after cultivation in various media, samples for RNA isolation were prepared as follows.

HeLa-PM-BFP cells were seeded at a density of 2.5 × 10^5^ in a 12-well plate and allowed to grow overnight. The following day, the medium was replaced with DMEM-2%FBS, and the cells were infected with *Bb bsp22*^SPOT^ / Δ*bteA* at MOI 100:1 followed by centrifugation (3 miutes, 300 *g*). Three hours post-infection at 37°C with 5% CO_2_, infected cells were lysed using 0.2% Triton X-100, which allowed the selective lysis of HeLa-PM-BFP cells while preserving bacterial integrity. The lysate was transferred into 1.5 mL Eppendorf tubes and centrifuged (10 minutes; 6,000 × *g*, 4°C). The supernatant was aspirated, and the bacterial pellet was lysed in prewarmed (65°C) TRI reagent (Merck, Cat# T9424) for RNA isolation.

For hNEC infection, differentiated ALI cultures were apically infected with 1 × 10^7^ CFU of *B. bronchiseptica* wild-type per Transwell membrane, using 100 µL of DMEM-2%FBS. To isolate RNA only from adherent bacteria, the medium containing non-adherent bacteria was carefully removed after the 3-hour incubation. The cells on the Transwell membranes were lysed using 0.33 mL of prewarmed (65°C) TRI reagent (Merck, Cat# T9424) per membrane, with lysis proceeding for 7 minutes. Homogenization was carried out by repeated pipetting to ensure complete cell disruption. Lysates from the three Transwell membranes were pooled to obtain sufficient material for RNA isolation.

To assess the effect of the infection medium (DMEM-2%FBS, Table S1), *B. bronchiseptica* strains grown overnight in *Bb*-SSM to OD_600_ ~1.5 (Table S1) were centrifuged (5 minutes; 6,000 *g*) and resuspended in either *Bb*-SSM or DMEM-2%FBS to an OD_600_ ~1.0. Bacteria were then cultivated at 37°C, 180 rpm for 3 hours, followed by centrifugation (5 minutes; 6,000 *g*). The bacterial pellet was lysed in prewarmed (65°C) TRI reagent (Merck, Cat# T9424) for RNA isolation.

RNA from TRI reagent samples was isolated using chloroform extraction that was repeated twice to achieve an optimal purity. Subsequently, RNA was precipitated by adding an equivalent volume of isopropanol, followed by incubation at −20°C for at least 45 minutes. RNA was pelleted for 45 minutes at 13,000 *g* at 4°C, and pellets were washed twice with freshly prepared 75% ethanol, briefly air-dried, and then resuspended in nuclease-free water. The RNA purity and concentration were determined using a DS-11 spectrophotometer DeNovix. The RNA samples were further cleaned using the TurboDNase Kit (Invitrogen, Cat# AM1907), following the manufacturer’s instructions. Reverse transcription was carried out using the High-Capacity cDNA Reverse Transcription Kit (ThermoFisher Scientific, Cat# 4368814), and quantitative PCR (qPCR) was performed using EvaGreen reagent (Solid Biodyne, Cat# 08-25-00001) with 20 ng of cDNA used per reaction. Data were collected using a CFX384 PCR instrument (BioRad). Two housekeeping genes, *rpoB* and *dnaA*, were used as reference genes. The sequences of the qPCR primers and their efficiencies are provided in Table S5; Fig. S10.

#### Determination of BteA^HiBiT^ injection into HeLa cells

Injection of BteA^HiBiT^ into HeLa cells was evaluated, as previously reported (23). In brief, 5 × 10^4^ of HeLa-LgBit (HeLa cells constitutively expressing LgBit) per well were seeded in 96-well white/clear-bottom plate (Corning) in DMEM-2%FBS. The next day, *B. bronchiseptica* reporter strains expressing BteA^HiBiT^ (Table S2) were washed in DMEM-2%FBS by centrifugation (5 minutes; 8,000 *g*) and added to cells at MOI 5:1 along with Nano-Glo Live Cell Reagent (Cat.No. N2011, Promega), containing cell-permeable luciferase substrate and Nano-Glo buffer. After centrifugation (5 minutes; 120 *g*), the plate was placed inside the chamber of TecanSpark microplate reader (Tecan) with 37°C and 5% CO_2_, and luminescence measurements were performed for 2 hours at 2-minute intervals.

#### Determination of bacterial CFUs and Bsp22 protein levels in infected ALI cultures of hNECs

Differentiated ALI cultures of hNECs were apically infected with 5 × 10^6^ CFU of *Bb bsp22*^HiBiT^ / Δ*bteA* per Transwell membrane, using 100 µL of DMEM-2%FBS, for durations of 3, 6, and 24 hours. To retrieve bacteria for determining CFUs and Bsp22 protein levels, 100 µL of PBS supplemented with 0.2% TX-100 (Merck, Cat# T8787) was added to the Transwell membrane, followed by a 5-minute incubation. The resulting suspension was then pipetted up and down and transferred into a tube, and an additional 100 µL of PBST was applied onto the Transwell membrane. After a 5-minute incubation, the suspension was once again pipetted up and down and transferred to the same tube. The removal of bacteria and epithelial cells from the Transwell membrane was confirmed by wide-field microscopy. For CFU determination, the suspension was plated at various dilutions on BG agar plates, and CFUs were counted after a 3-day incubation period. To assess Bsp22 protein levels, the Nano-Glo HiBiT Extracellular Detection System (Promega, Cat# N2420) was utilized following the manufacturer’s instructions. In brief, suspension (diluted 1:10) was mixed with recombinant LgBit protein, furimazine substrate, and Nano-Glo buffer. Luminescence was subsequently measured using the TecanSpark microplate reader (Tecan).

#### Graph preparation and statistical analysis

Prism9 (GraphPad, https://www.graphpad.com/) was used for graph preparation and statistical analysis.

## Data Availability

The custom code for quantitative analysis of Bsp22 filaments is available on https://github.com/LMCF-IMG/T3SS-filaments. Individual filament length measurements are provided within the figures and supplementary materials of this manuscript.

## SUPPLEMENTAL MATERIAL

Supplemental Figures mbio.01135-25-s0001.pdf Fig. S1 to S10.

Supplemental Tables mbio.01135-25-s0002.pdf Tables S1 to S5.

ASM does not own the copyrights to Supplemental Material that may be linked to, or accessed through, an article. The authors have granted ASM a non-exclusive, world-wide license to publish the Supplemental Material files. Please contact the corresponding author directly for reuse.
