# Supplementary material for: Assembly and dynamic regulation of the tip filament of the Bordetella type III secretion system injectisome
Source: mBio. 2025 Sep 22;16(11):e01135-25. doi: 10.1128/mbio.01135-25 (PMC12607632; doi:10.1128/mbio.01135-25)
Supplement: Supplemental Figures — Fig. S1 to S10. [file mbio.01135-25-s0001.pdf]

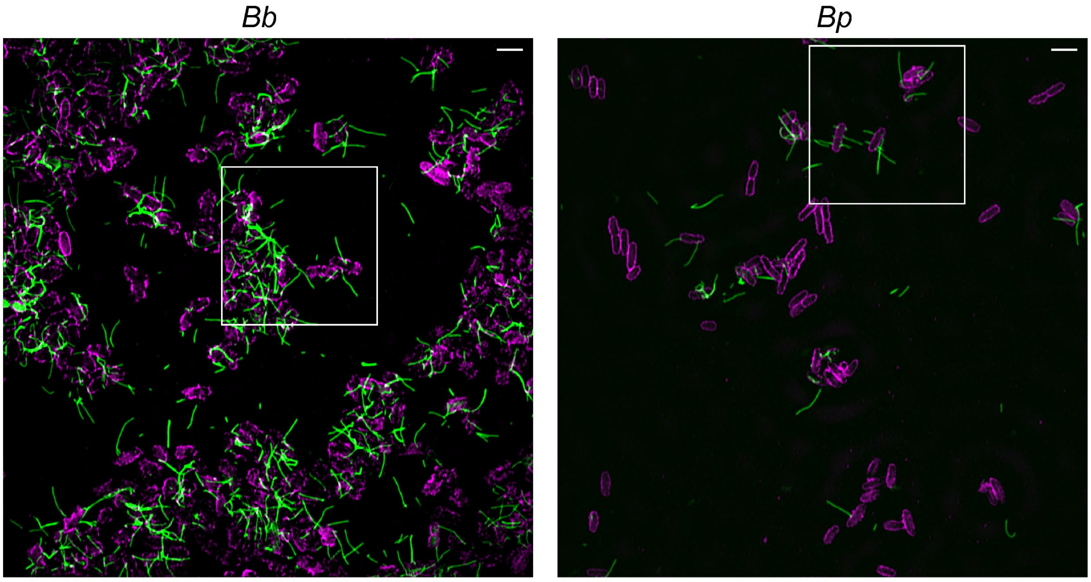

Figure S1. The Bsp22 protein polymerizes into flexible filaments that intertwine across the surface of a glass coverslip (related to Fig. 1).

Cells of *B. bronchiseptica* RB50 (*Bb*) or *B. pertussis* B1917 (*Bp*) expressing Bsp22^{SPOT} were centrifugated onto glass coverslips and incubated for 3 h in *Bb*-SSM or *Bp*-SSM, respectively. After fixation, Bsp22 (green) was visualized with the Spot-label ATTO488, while the bacterial cell surface (magenta) was labeled with a rabbit anti-*Bordetella* serum followed by the anti-rabbit IgG-DyLight 405 conjugate. The white squares indicate the regions magnified in Fig. 1C. The presented SIM images depict a single focal plane and are representative of three independent experiments. Scale bar, 2 µm.

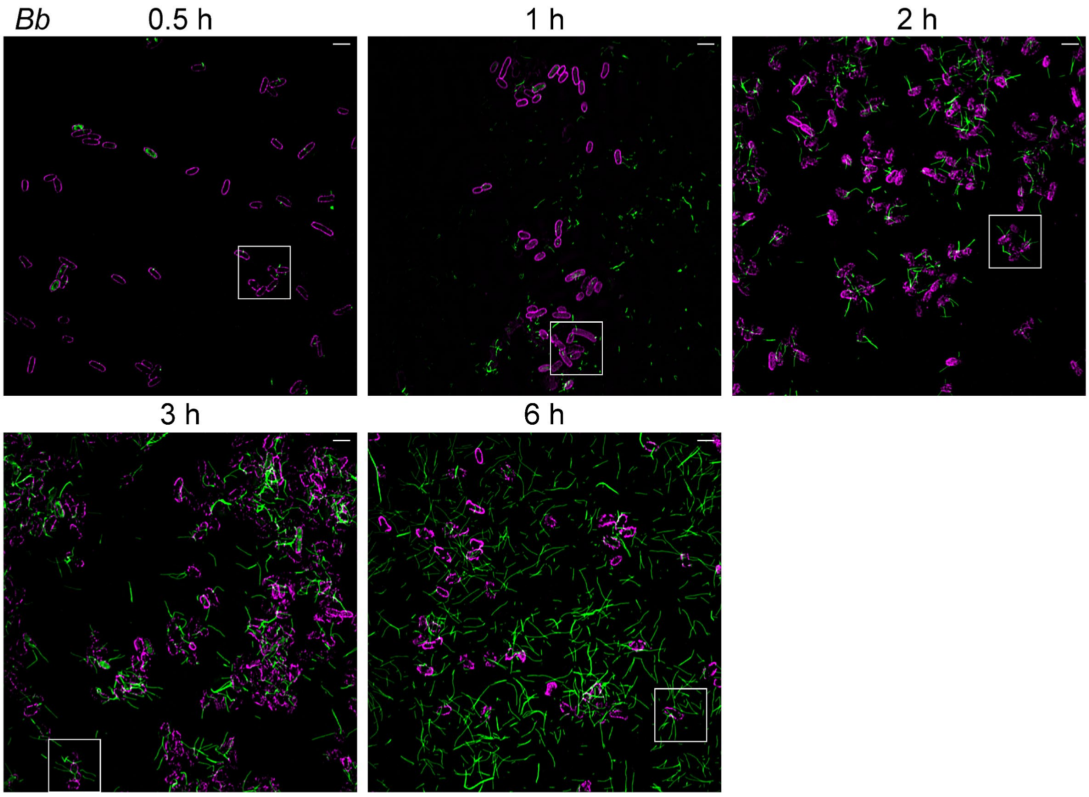

Figure S2. The Bsp22 filaments elongate on glass coverslips (related to Fig. 2).

Cells of *Bb bsp22*^{SPOT} incubated on glass coverslips in *Bb*-SSM were fixed at the indicated time points, and stained as described in the legend of Fig. S1. Bsp22, green; bacterial cell surface, magenta. The white squares indicate regions magnified in Fig. 2A. The SIM images show a single focal plane and are representative of three independent experiments. Scale bars, 2 µm.

A

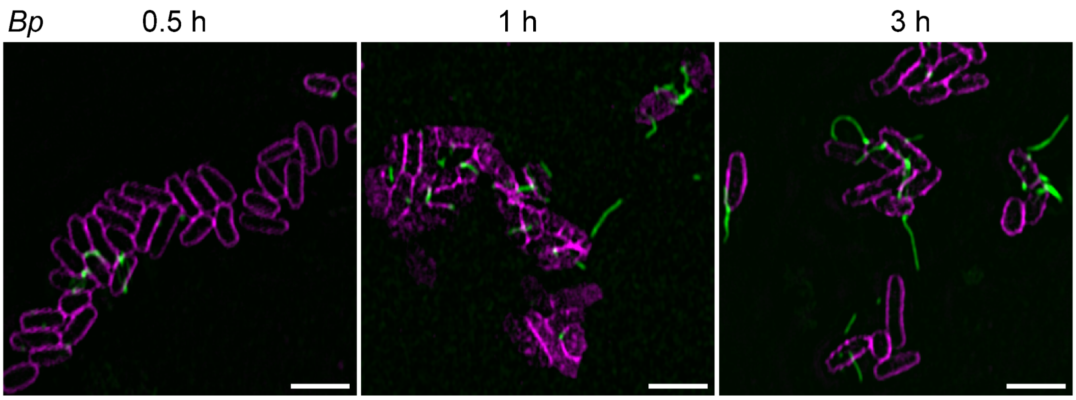

B

SSM

Time (h)	Cells analyzed	Cells with filaments (%)	Filaments per cell if present	Filament length (μm)
0.5	138	71 ± 2.9	1.9 ± 0.61	0.1 ± 0.09
1	161	61 ± 7.3	2.1 ± 0.18	0.5 ± 0.50
3	93	45 ± 3.3	1.9 ± 0.31	0.8 ± 0.71

C

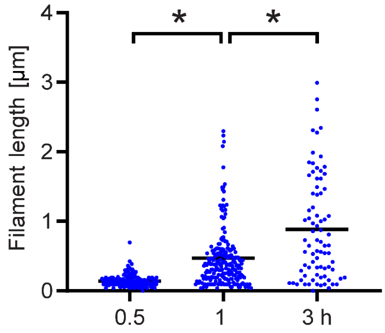

Figure S3. The Bsp22 filaments of *B. pertussis* also elongate on glass coverslips (related to Fig. 2).

(A) Visualization of Bsp22 filaments at different time points. Cells of *Bp bsp22*^{SPOT} were incubated on glass coverslips in *Bp*-SSM, fixed at indicated time points, and stained as described in the legend of Fig. S1. Bsp22, green; bacterial cell surface, magenta. Images are single focal planes and representative of three independent experiments. Scale bars, 2 μm.

(B-C) Quantitative analysis of Bsp22 filaments in *B. pertussis* at different time points. (B) The percentage of *Bp bsp22*^{SPOT} cells with filaments, filament count per cell, and filament length were determined using a custom-made macro for Fiji. Data are presented as mean ± SD, calculated from multiple microscopy fields in a single representative experiment. (C) Dot plot of filament lengths. The black bar indicates the mean. *$p < 0.05$, unpaired two-tailed t-test.

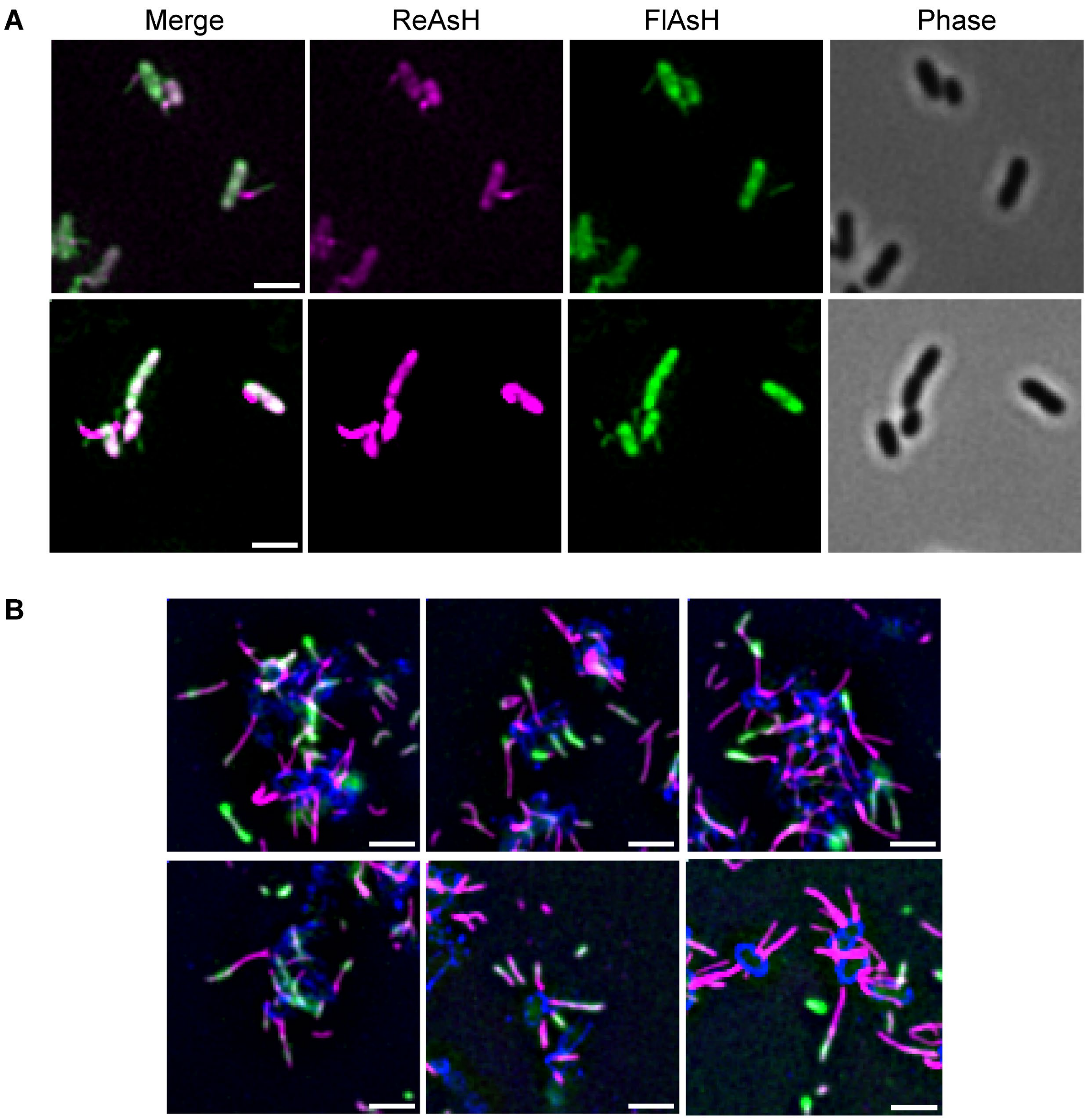

Figure S4. Bsp22 monomers are added at the distal filament end (related to Fig. 3).

(A) Cells of *Bb bsp22*^{TC} were sequentially labeled with ReAsH (magenta) followed by FlAsH (green) in a pulse-chase experiment and visualized under a wide-field microscope (Olympus). ReAsH was followed in the TexasRed channel and FlAsH in the FITC channel using specific excitation and emission filters. Scale bar, 2 μm.

(B) Cells of *Bb bsp22*^{SPOT} carrying a plasmid for inducible expression of Bsp22^{ALFA} were seeded on coverslips in *Bb*-SSM. After 3 hours, Bsp22^{ALFA} expression was induced by 1 mM IPTG, while chromosomal Bsp22^{SPOT} expression was attenuated by adding 50 mM $MgSO_4$. Following 5 hours, cells were fixed and stained. Bsp22-SPOT (magenta) was visualized using Spot-label ATTO594, Bsp22-ALFA (green) was detected using an ALFA-ATTO488 nanobody, and the bacterial cell surface (blue) was labeled with rabbit anti-*Bordetella* serum, followed by anti-rabbit IgG-DyLight405 conjugate. Images were acquired using a wide-field microscope (Olympus). Scale bar, 2 μm.

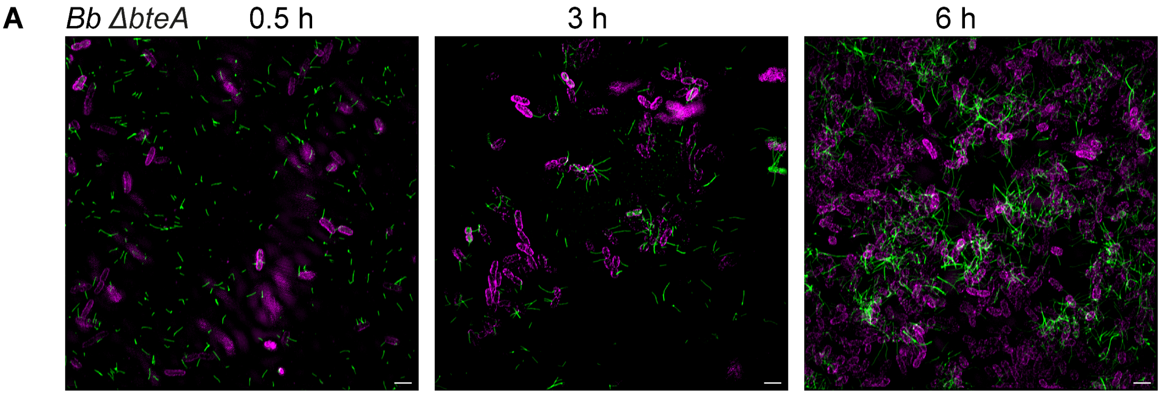

B

SSM

Time (h)	Cells analyzed	Cells with filaments (%)	Filaments per cell if present	Filament length (µm)
0.5	367	67 ± 2.6	1.8 ± 0.14	0.5 ± 0.42
3	390	52 ± 0.2	1.7 ± 0.08	1.0 ± 0.81
6	455	55 ± 1.6	1.8 ± 0.12	1.1 ± 0.73

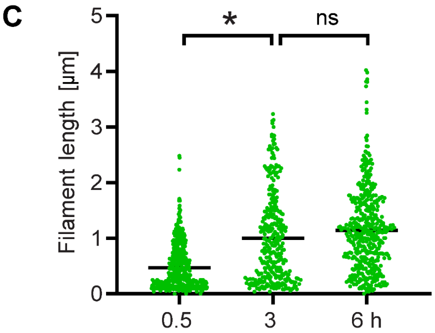

Figure S5. Absence of the BteA effector does not hinder Bsp22 filament formation and elongation (related to Fig. 4).

(A) Visualization of Bsp22 filaments at different time points. Cells of *Bb bsp22*^{SPOT}/ Δ*bteA* cells were incubated on coverslips in *Bb*-SSM, fixed at the indicated time points, and stained as described in the legend of Fig. S1. Bsp22, green; bacterial cell surface, magenta. Images are single focal planes and representative of three independent experiments. Scale bars, 2 µm.

(B-C) Quantitative analysis of Bsp22 filaments. (B) The percentage of *Bb bsp22*^{SPOT}/ Δ*bteA* cells with filaments, filament count per cell, and filament length were determined at different time points in a representative experiment. Data were processed using a custom-made macro for Fiji, and are presented as mean ± SD from multiple microscopy fields. (C) Dot plot of filament lengths. The black bar indicates the mean. *$p < 0.05$, unpaired two-tailed t-test.

A

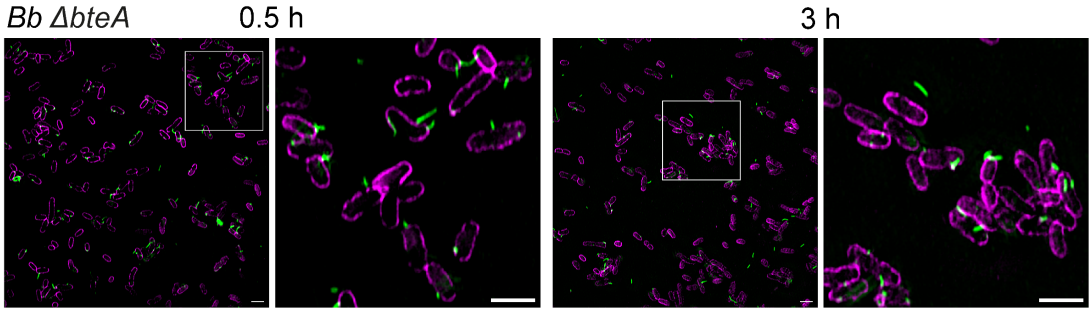

B

DMEM

Time (h)	Cells analyzed	Cells with filaments (%)	Filaments per cell if present	Filament length (µm)
0.5	545	56 ± 1.1	2.2 ± 0.61	0.3 ± 0.28
3	908	46 ± 5.1	1.5 ± 0.08	0.3 ± 0.36

C

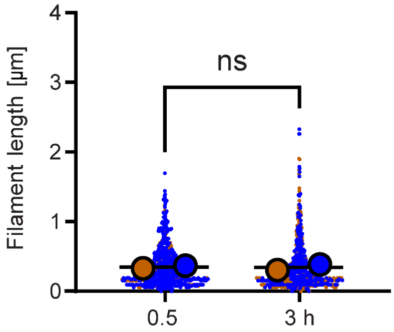

Figure S6. Visualization of Bsp22 filaments on glass coverslips in DMEM medium (related to Fig. 4).

(A) Imaging of Bsp22 filaments on glass coverslips in DMEM medium. Cells of *Bb bsp22*^{SPOT}/ Δ*bteA* cells were incubated on coverslips in DMEM-2%FBS (DMEM), fixed at the indicated time points, and stained as described in the legend of Fig. S1. Bsp22, green; bacterial cell surface, magenta. Images are single focal planes and representative of three independent experiments. Scale bars, 2 µm.

(B-C) Quantitative analysis of Bsp22 filaments. (B) The percentage of *Bb bsp22*^{SPOT}/ Δ*bteA* cells with filaments, filament count per cell, and filament length were determined from cells cultivated on glass coverslips in DMEM-2%FBS (DMEM) in two independent experiments. Data are presented as mean ± SD from multiple microscopy fields. (C) SuperPlots of filament lengths from two independent experiments. Distinct colors represent each experiment, with circles indicating the mean filament length of each experiment. The black bar shows the overall average. *$p < 0.05$, unpaired two-tailed t-test. Please, note that data from 3 h time points were used also in Fig. 4D.

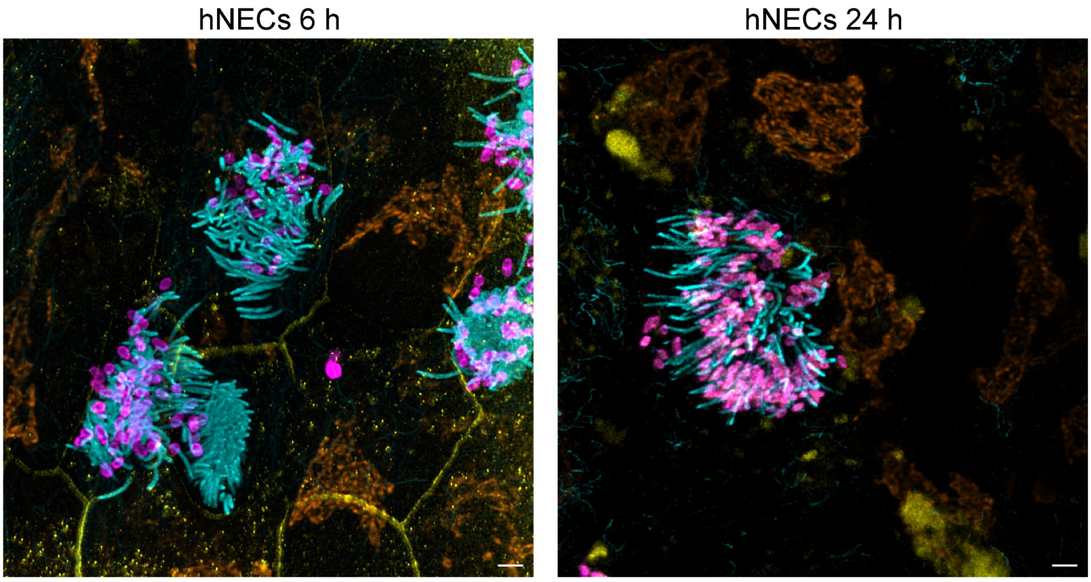

Figure S7. Visualization of *B. bronchiseptica* on hNECs.

Representative images of hNECs infected with the *B. bronchiseptica* Δ*bteA* derivative expressing the mScarlet fluorescence protein (*Bb* Δ*bteA* // mSc) at 6 and 24 h post-infection. The surface of hNECs was stained with wheat germ agglutinin (WGA, orange) conjugated to AF647 (5 µg/ml, 15 min, RT). The tight junction protein ZO-1 (yellow) was stained with a rabbit-anti-ZO-1 antibody followed by anti-rabbit IgG-DyLight 405. Disruptions in the ZO-1 network are visible due to the *B. bronchiseptica* colonization. Cilia (cyan) were stained with a mouse anti-acetylated tubulin antibody, visualized by anti-mouse IgG-AF488. *B. bronchiseptica* cells (magenta) were visualized by mScarlet expression. Z-MAX projections are shown. Scale bars, 2 µm.

Confocal images were acquired with Leica STELLARIS 8 equipped with a wide-range (440 – 790 nm) light laser with the pulse picker (WLL PP) and highly sensitive hybrid detectors operated by the LAS X software. The objective HC PL APO 40x/1.25 GLYC CORR CS2, WD 0.35 mm, was used with the type G immersion (Leica).

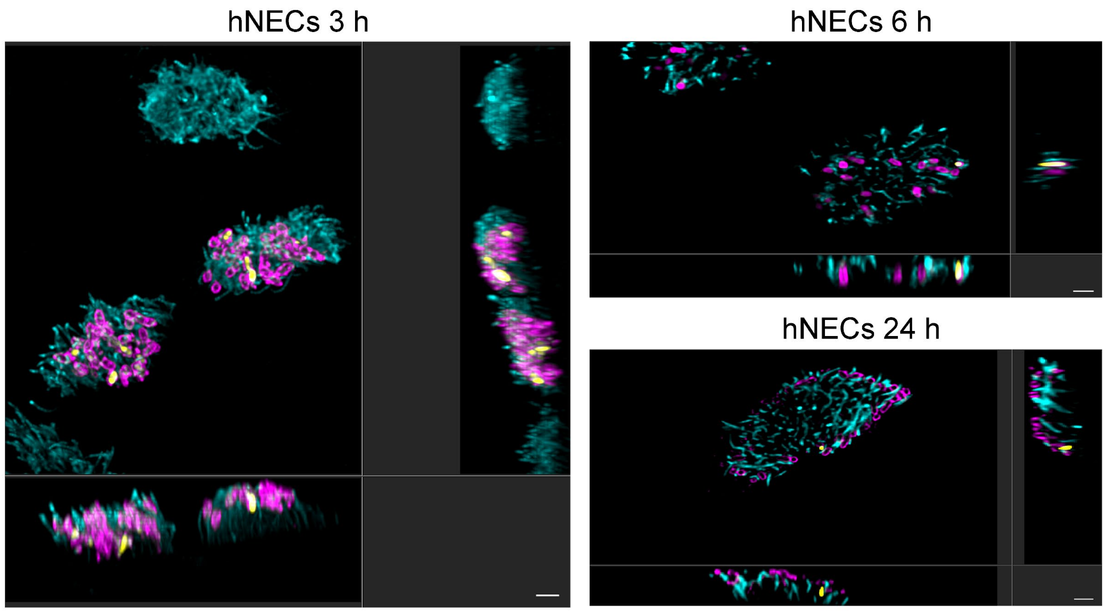

Figure S8. Bsp22 filaments become scarce during *B. bronchiseptica* infection of hNECs (related to Fig. 5).

Representative images of apical surfaces of hNECs infected with *Bb bsp22*^{SPOT}/ Δ*bteA*. Bsp22 filaments (yellow), bacterial cell surface (magenta), and cilia (cyan) were stained as described in Materials and Methods. Orthogonal views are representative of three independent experiments. Scale bars, 2 μm.

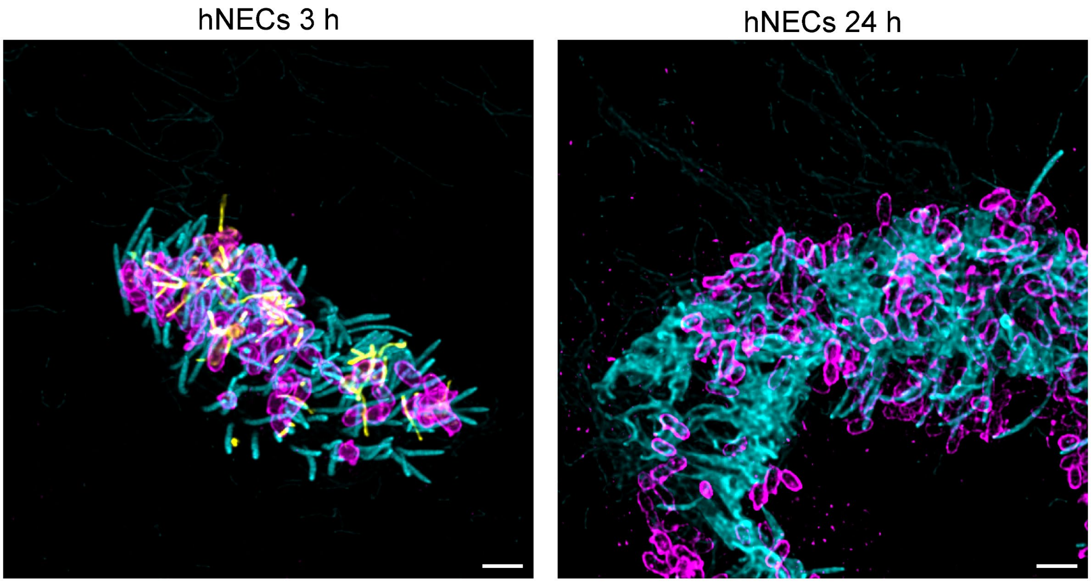

Figure S9. Bsp22 filaments become scarce during *B. bronchiseptica* infection of hNECs (related to Fig. 5).

Representative images of apical surfaces of hNECs infected with *Bb bsp22*^{SPOT}/ Δ*bteA* document that even after using a different mode of hNEC infection, Bsp22 filaments become scarce in the course of infection. In this infection method, 3 × 10^6 bacterial cells were distributed in five 1-µl drops of SSM medium over the surface of the Transwell membrane with hNECs cultured at ALI. *Bb bsp22*^{SPOT}/ Δ*bteA* was cultured overnight in *Bb*-SSM medium to the exponential phase before infection. Bsp22 (yellow), bacterial cell surface (magenta), and cilia (cyan) were stained as described in as described in Materials and Methods. Z-MAX projections are shown. Scale bars, 2 µm.

Confocal images were acquired with Leica STELLARIS 8 equipped with a wide-range (440 – 790 nm) light laser with the pulse picker (WLL PP) and highly sensitive hybrid detectors operated by the LAS X software. The objective HC PL APO 40x/1.25 GLYC CORR CS2, WD 0.35 mm, was used with the type G immersion (Leica).

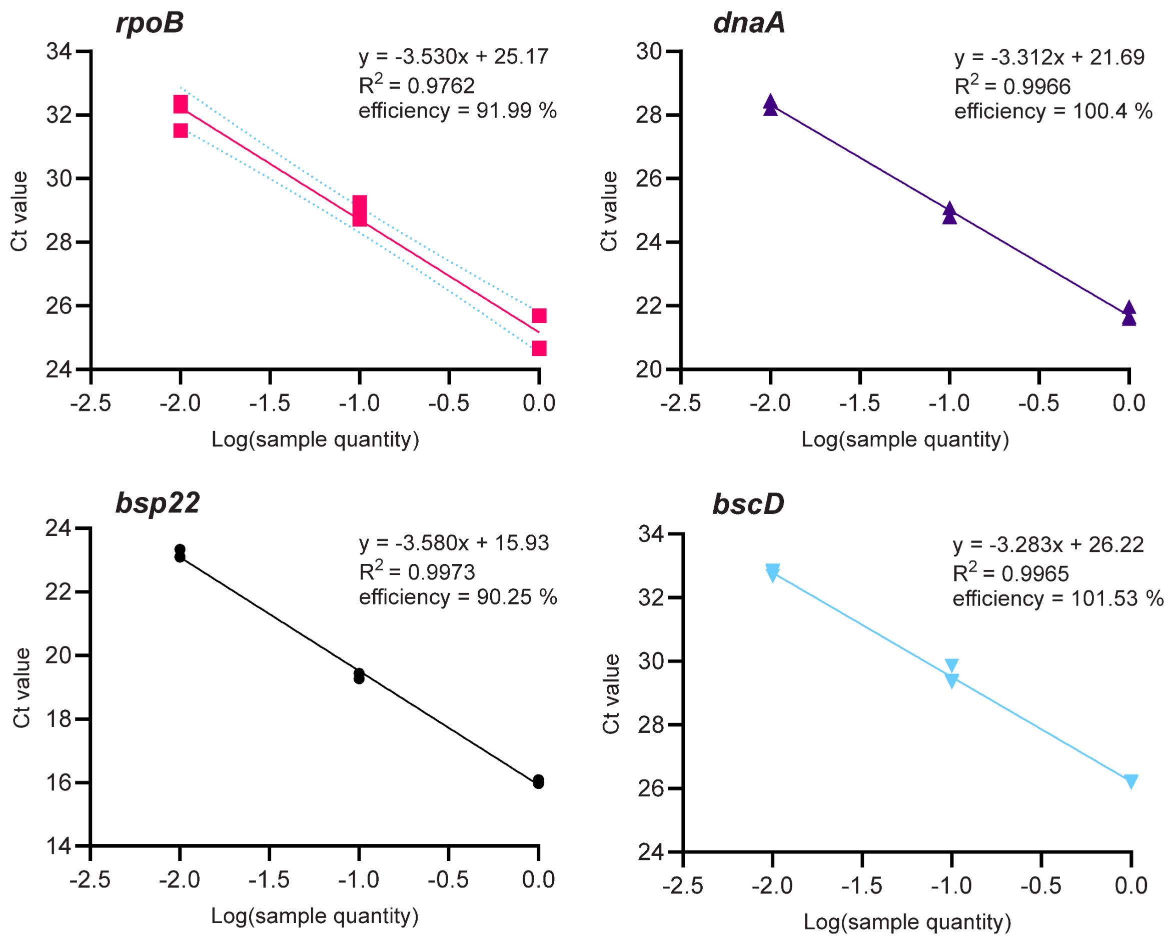

Figure S10. Determination of qRT-PCR primer amplification efficiency.

The amplification efficiency of qRT-PCR primers for *rpoB*, *dnaA*, *bsp22*, and *bscD* genes was determined using standard curve analysis. Standard curves were generated by plotting the mean CT values against the corresponding serial dilutions of cDNA.
