# Supplementary material for: Assembly and dynamic regulation of the tip filament of the Bordetella type III secretion system injectisome
Source: mBio. 2025 Sep 22;16(11):e01135-25. doi: 10.1128/mbio.01135-25 (PMC12607632; doi:10.1128/mbio.01135-25)
Supplement: Supplemental Tables — Tables S1 to S5. [file mbio.01135-25-s0002.pdf]

**Malcova *et al.*, Suppl. tables**

**S1 Table. COMPOSITION OF CELL CULTURE AND BACTERIAL CULTIVATION MEDIA**

| CHEMICAL | SOURCE | IDENTIFIER / Cat. No. | FINAL CONC. |
|---|---|---|---|
| **DMEM-PhenolRed-10%FBS for HeLa cells and HeLa-Rpre-BFP** | | | |
| DMEM | Sigma-Aldrich/Merck | Cat# D6429 | 1x |
| Fetal Bovine Serum | Gibco | Cat# 10270-106 | 10 % |
| **DMEM-10%FBS** | | | |
| DMEM-noPhenolRed | Institute of Molecular Genetics, Prague, CZ | Cat# N/A | 1x |
| Fetal Bovine Serum | Gibco | Cat# 10270-106 | 10 % |
| **DMEM-2%FBS** | | | |
| DMEM-noPhenolRed | Institute of Molecular Genetics, Prague, CZ | Cat# N/A | 1x |
| Fetal Bovine Serum | Gibco | Cat# 10270-106 | 2 % |
| **DMEM medium for 3T3-J2 cell line** | | | |
| DMEM | Sigma-Aldrich/Merck | Cat# D6429 | |
| Bovine Calf Serum | Sigma-Aldrich/Merck | Cat# 12138C | 10% |
| Penicillin-Streptomycin | Gibco | Cat# 15070-063 | 1 % |
| **NEC (nasal epithelial cell) medium** | | | |
| DMEM | Sigma-Aldrich/Merck | Cat# D6429 | 60 % |
| F12 | Gibco | Cat# 11765-054 | 30 % |
| Fetal Bovine Serum (charcoal stripped) | Sigma-Aldrich/Merck | Cat# F6765 | 7.5 % |
| Penicillin-Streptomycin | Gibco | Cat# 15070-063 | 1 % |
| Insulin | Sigma-Aldrich/Merck | Cat# I9278 | 5 µg /ml |
| Epidermal Growth Factor | PromoCell Suppl. Pack | Cat# C-39170 | 0.125 ng /ml |
| Hydrocortisone | PromoCell Suppl. Pack | Cat# C-39170 | 25 ng /ml |
| Cholera toxin | Sigma-Aldrich/Merck | Cat# C-8052 | 8.6 ng /ml |
| Y-27632 dihydrochloride | Tocris | Cat# 1254 | 5 µM |
| Fungin | Invivogen | Cat# Ant-fn-1 | 10 µg /ml |
| **ALI (air-liquid interface) medium** | | | |
| DMEM | Sigma-Aldrich/Merck | Cat# D6429 | 50 % |
| LHC Basal | Invitrogen | Cat# 12677-019 | 50 % |
| $NaSeO_3$ | Sigma-Aldrich/Merck | Cat# S5261 | 30 µM |
| $MnCl_2.4H_2O$ | Sigma-Aldrich/Merck | Cat# M5005 | 1 µM |
| $Na_2SiO_3$.9H2O | Sigma-Aldrich/Merck | Cat# S5904 | 0.5 µM |
| $(NH_4)_6Mo_7O_{24}.{}_4H_2O$ | Sigma-Aldrich/Merck | Cat# M1019 | 1 µM |
| $NH_4VO_3$ | Sigma-Aldrich/Merck | Cat# 398128 | 5 µM |
| $NiSO_4.6H_2O$ | Sigma-Aldrich/Merck | Cat# N4882 | 1 µM |
| $SnCl_2.2H_2O$ | Sigma-Aldrich/Merck | Cat# S9262 | 0.5 µM |
| $ZnSO_4.7H_2O$ | Sigma-Aldrich/Merck | Cat# Z0251 | 3 µM |
| $FeSO_4.7H_2O$ | Sigma-Aldrich/Merck | Cat# F8633 | 1.5 µM |
| $MgCl_2.6H_2O$ | Sigma-Aldrich/Merck | Cat# M2670 | 0.6 mM |
| $CaCl_2.2H_2O$ | Penta | Cat# 16790-31000 | 0.11 mM |
| Phosphorylethanolamine | Sigma-Aldrich/Merck | Cat# P0503 | 0.5 µM |
| Ethanolamine | Sigma-Aldrich/Merck | Cat# E0135 | 0.5 µM |
| Retinoic Acid | Sigma-Aldrich/Merck | Cat# R2625 | 0.05 µM |
| Bovine Serum Albumin | PromoCell Suppl. Pack | Cat# C-39170 | 0.5 mg/ml |
| Insulin | PromoCell Suppl. Pack | Cat# C-39170 | 5 µg/ml |

| | | | |
|---|---|---|---|
| Triiodo-L-thyronine | PromoCell Suppl. Pack | Cat# C-39170 | 6.7 ng/ml |
| Transferrin | PromoCell Suppl. Pack | Cat# C-39170 | 10 µg/ml |
| Epinephrine | PromoCell Suppl. Pack | Cat# C-39170 | 0.5 µg/ml |
| Bovine Pituitary Extract | PromoCell Suppl. Pack | Cat# C-39170 | 10 µg/ml |
| Hydrocortisone | PromoCell Suppl. Pack | Cat# C-39170 | 76 ng/ml |
| EGF | PromoCell Suppl. Pack | Cat# C-39170 | 0.5 ng/ml |
| Penicilin-Streptomycin | Gibco | Cat# 15070-063 | 2 % |
| **Modified Stainer-Scholte medium for *B. bronchiseptica* (*Bb*-SSM)** | | | |
| L-glutamate (monosodium salt) | Sigma-Aldrich/Merck | Cat# 49621 | 11.5 mM |
| L-proline | Sigma-Aldrich/Merck | Cat# P5607 | 2.1 mM |
| NaCl | Lach:ner | Cat# 30093-AP0 | 42.8 mM |
| $KH_2PO_4$ | Lach:ner | Cat# 30060-AP0 | 3.7 mM |
| KCl | Lach:ner | Cat# 30076-CP0 | 2.7 mM |
| $MgCl_2.6H_2O$ | Sigma-Aldrich/Merck | Cat# M2670 | 0.5 mM |
| Tris HCl | Serva | Cat# 37192.02 | 40.4 mM |
| Tris base | Sigma-Aldrich/Merck | Cat# 10708976001 | 9.7 mM |
| L-Cystein hydrochloride monohydrate | Sigma-Aldrich/Merck | Cat# 30129 | 330 µM |
| Ascorbic acid | Sigma-Aldrich/Merck | Cat# A92902 | 110 µM |
| Nicotinic acid (Niacin) | Sigma-Aldrich/Merck | Cat# NO761 | 32.5 µM |
| Glutathione | Roth | Cat# 6382.2 | 32.5 µM |
| Casamino acids | Difco | Cat# 223050 | 5 g/ l |
| **Modified Stainer-Scholte medium for *B. pertussis* (*Bp*-SSM)** | | | |
| L-glutamate (monosodium salt) | Sigma-Aldrich/Merck | Cat# 49621 | 11.5 mM |
| L-proline | Sigma-Aldrich/Merck | Cat# P5607 | 2.1 mM |
| NaCl | Lach:ner | Cat# 30093-AP0 | 42.8 mM |
| $KH_2PO_4$ | Lach:ner | Cat# 30060-AP0 | 3.7 mM |
| KCl | Lach:ner | Cat# 30076-CP0 | 2.7 mM |
| $MgCl_2.6H_2O$ | Sigma-Aldrich/Merck | Cat# M2670 | 0.5 mM |
| $CaCl_2.2H_2O$ | Penta | Cat# 16790-31000 | 0.1 mM |
| Tris HCl | Serva | Cat# 37192.02 | 40.4 mM |
| Tris base | Sigma-Aldrich/Merck | Cat# 10708976001 | 9.7 mM |
| L-Cysteine hydrochloride monohydrate | Sigma-Aldrich/Merck | Cat# 30129 | 330 µM |
| Ascorbic acid | Sigma-Aldrich/Merck | Cat# A92902 | 110 µM |
| Nicotinic acid (Niacin) | Sigma-Aldrich/Merck | Cat# NO761 | 32.5 µM |
| Glutathione | Roth | Cat# 6382.2 | 32.5 µM |
| Casamino acids | Difco | Cat# 223050 | 5 g/ l |
| Cyklodextrin | Merck | Cat# W402826 | 1 mg/ ml |
| **reducing *Bordetella* labelling medium (BLM)** | | | |
| L-glutamate (monosodium salt) | Sigma-Aldrich/Merck | Cat# 49621 | 11.5 mM |
| L-Cysteine hydrochloride monohydrate | Sigma-Aldrich/Merck | Cat# 30129 | 330 µM |
| Ascorbic acid | Sigma-Aldrich/Merck | Cat# A92902 | 110 µM |
| Nicotinic acid (Niacin) | Sigma-Aldrich/Merck | Cat# NO761 | 32.5 µM |
| Glutathione | Roth | Cat# 6382.2 | 32.5 µM |
| $NaHPO_4.12H_2O$ | Lach:ner | Cat# 30061-CPO | 42 mM |
| $KH_2PO_4$ | Lach:ner | Cat# 30060-AP0 | 22 mM |
| NaCl | Lach:ner | Cat# 30093-AP0 | 8.6 mM |
| $NH_4Cl$ | Lach:ner | Cat# 30070-CP0 | 18.6 mM |

| | | | |
|---|---|---|---|
| $CaCl_2.2H_2O$ | Penta | Cat# 16790-31000 | 68 µM |
| $MgSO_4.7H_2O$ | Sigma-Aldrich/Merck | Cat# M1880 | 810 µM |
| $FeSO_4.7H_2O$ | Sigma-Aldrich/Merck | Cat# 215422 | 5.4 µM |
| TCEP | Sigma-Aldrich/Merck | Cat# 646547 | 0.5 mM |

**S2 Table. DESCRIPTION OF BACTERIAL STRAINS USED IN THIS STUDY.**

| STRAIN | GENOTYPE AND DESCRIPTION | REFERENCE |
|---|---|---|
| ***Esterichia coli* strains** | | |
| XL1-Blue | *rec*A1 *end*A1 *gyr*A96 *thi*-1 *hsd*R17 *sup*E44 *rel*A1 *lac* F´ *pro*AB *lacIq*ZΔM15 Tn10 Tet$^r$ | Stratagene |
| SM10 λpir | *thi thr leu ton*A *lac*Y *sup*E *rec*A::RP4-2-Tc::Mu Km λpir | (1, 2) |
| BL21 λ(DE3) | F– *omp*T *hsd*$S_B$ (rB–, mB–) *gal dcm* (DE3) | Novagen |
| ***Bordetella bronchiseptica* strains** | | |
| *Bb* WT | *Bb*RB50 WT; wild type *Bordetella bronchiseptica* RB50 (B1976); complex I rabbit isolate; ST-12 | (3), laboratory stock ID BB012 |
| *Bb ΔbteA* | *BbRB50 ΔbteA*; *Bb*RB50 strain derivative with *bte*A in-frame deletion of codons L2-A657 | (4), laboratory stock ID BB020 |
| *Bb Δbsp22* | *BbRB50 Δbsp22*; *Bb*RB50 strain derivative with *bsp22* in-frame deletion of codons T10-S198 | This study, laboratory stock ID BB212 |
| *Bb bsp22*$^{SPOT}$ | *Bb* bsp22$^{SPOT}$; *BbRB50* strain derivative encoding *bsp22* allele with insertion of SPOT-tag flanked by GSSG linkers (GSSG**PDRVRAVSHWSS**GSSG) in between codons M138 and A139 | This study, laboratory stock ID BB237 |
| *Bb bsp22*$^{SPOT}$ /Δ*bteA* | *Bb bsp22*$^{SPOT}$ / Δ*bteA; Bb bsp22*$^{SPOT}$ strain derivative with *bteA* in-frame deletion of codons L2-A657 | This study, laboratory stock ID BB175 |
| *Bb bsp22*$^{TC}$ | *Bb* bsp22$^{TC}$; *BbRB50* strain derivative encoding *bsp22* allele with insertion of TC-tag flanked by GSSG linkers (GSSG**FLNCCPGCCMEP**GSSG) in between codons M138 and A139 | This study, laboratory stock ID BB127 |
| *Bb bsp22*$^{SPOT}$ // *bsp22*$^{ALFA}$ | *Bb bsp22*$^{SPOT}$// pBBRI-*lacIq-Ptac-bsp22*$^{ALFA}$, *Bb bsp22*$^{SPOT}$ harboring pBBRI-encoded repressor LacIq and Bsp22 with insertion of ALFA-tag flanked by GSSG linkers (GSSG**SRLEEELRRRLTE**GSSG) in between codons M138 and A139 under the control of Ptac promoter | This study, Laboratory stock ID pBB147 |
| *Bb bteA*$^{HiBiT}$ | *Bb*RB50 WT encoding *bteA* codons M1-V130 fused to HiBiT with GSSG linker at C-terminus, *bteA* codons K131-A657 were removed, STOP codon is retained | (4), Laboratory stock ID BB034 |
| *Bb bteA*$^{HiBiT}$ / *Δbsp22* | *Bb bteA*$^{HiBiT}$ derivative with *bsp22* in-frame deletion of codons T10-S198 | (4), Laboratory stock ID BB057 |
| *Bb bteA*$^{HiBiT}$ / *Δbsp22 // bsp22* | *Bb bteA*$^{HiBiT}$ */ Δbsp22* derivative harboring pBBRI-encoded *bsp22* allele of *Bb*RB50 under native *bsp22* promoter (Pbsp22) | This study, Laboratory stock ID pBB195 |
| *Bb bteA*$^{HiBiT}$/ *ΔbscN* | *bteA*$^{HiBiT}$ derivative with *bscN* in-frame deletion of codons R2-E443 | (4), Laboratory stock ID BB051 |
| *Bb ΔbteA // mSc* | *BbRB50 ΔbteA* derivative harboring pBBRI-encoded mScarlet (mSc) under the control of *Bp*TohamaI BvgAS-regulated filamentous hemagglutinin (*fhaB* gene) promoter (PfhaB) | (5), Laboratory stock ID pBB117 |

| | | |
|---|---|---|
| *Bb bsp22^HiBiT /ΔbteA* | *Bb bsp22^HiBiT /ΔbteA; BbRB50 ΔbteA* strain derivative encoding *bsp22* allele with insertion of HiBiT-tag flanked by GSSG linkers (GSSGVSGWRLFKKISGSSG) in between codons M138 and A139 | This study, laboratory stock ID BB244 |
| ***Bordetella pertussis* strains** | | |
| *Bp* WT | *Bp*B1917 WT; wild type *Bordetella pertussis* B1917*; fim2-1, fim3-2, ptxP3, ptxA1, ptxB2, ptxC2, ptxD1, ptxE1, prn2* | (6, 7), laboratory stock ID BP001 |
| *Bp bsp22^SPOT* | *Bp bsp22^SPOT*; *Bp*B1917 strain derivative encoding *bsp22* allele with insertion of SPOT-tag flanked by GSSG linkers (GSSG**PDRVRAVSHWSS**GSSG) in between codons M138 and A139 | This study, laboratory stock ID BP082 |

**S3 Table. DESCRIPTION OF PLASMIDS USED IN THIS STUDY.**

| PLASMID | DESCRIPTION | REFERENCE |
|---|---|---|
| pSS4245 | Allelic exchange vector for *Bordetella spp.*, contains *ptx* promoter*, I-SceI*, *oriV*, *AmpR, StrR, KmR, BleR, TetR* and an *I-SceI* cleavage site for counterselection | (8, 9) |
| pSS4245 *Bb Δbsp22* | pSS4245 vector containing *BbRB*50 homology regions h1 (769 bp, 1721541-1722309) and h2 (740 bp, 1722877-1723616) flanking in-frame deletion of codons T10-S198 of Bsp22 | This study |
| pSS4245 *Bb bsp22^SPOT* | pSS4245 vector containing *BbRB*50 homology regions h1 (665 bp, 1722032-1722696) and h2 (709 bp, 1722697-1723405) flanking codons M138 and A139 of Bsp22, with inserted sequence encoding the SPOT-tag surrounded by GSSG linkers | This study |
| pSS4245 *Bb*RB50 Δ*bteA* | pSS4245 vector containing *BbRB*50 homology regions h1 (712 bp, 4501345-4502056) and h2 (622 bp, 4504028-4504649) flanking in-frame deletion of codons L2-A657 in the *bteA* of *Bb*RB50 | (4) |
| pSS4245 *Bb bsp22^TC* | pSS4245 vector containing *BbRB*50 homology regions h1 (665 bp, 1722032 to 1722696) and h2 (709 bp, 1722697 to 1723405) flanking codons M138 and A139 of Bsp22, with inserted sequence encoding the TC-tag surrounded by GSSG linkers | This study |
| pSS4245 *Bb bsp22^HiBiT* | pSS4245 vector containing *BbRB*50 homology regions h1 (665 bp, 1722032 to 1722696) and h2 (709 bp, 1722697 to 1723405) flanking codons M138 and A139 of Bsp22, with inserted sequence encoding the HiBiT-tag surrounded by GSSG linkers | This study |
| pSS4245 *Bp bsp22^SPOT* | pSS4245 vector containing *Bp*B1917 homology regions h1 (414 bp, 2292444-2292031) and h2 (708 bp, 2292030-2291323) flanking codons M138 and A139 of Bsp22, with inserted sequence encoding the SPOT-tag surrounded by GSSG linkers | This study |
| pBBRI MCS | *lacPOZ' mob^+*, broad-host cloning vector, $Cm^R$ | (10, 11) |

| | | |
|---|---|---|
| pBBRI-*lacIq*-Ptac-*bsp22*$^{ALFA}$ | pBBRI vector encoding the repressor LacIq and Bsp22 with insertion of ALFA-tag flanked by GSSG linkers in between codons M138 and A139 under the control of promoter Ptac | This study |
| pBBRI-Pbsp22-*bsp22* | pBBRI vector encoding the Bsp22 of *Bb*RB50 under the control its native promoter (Pbsp22) | This study |
| pUC18T-miniTn7T-gm-lacIq-Ptac | miniTn7 delivery plasmid with lacIq-Ptac inducible promoter, Addgene item #110558 | (12) |
| R-pre-GFP | Mammalian expression plasmid encoding plasma membrane target GFP, Addgene item #17274 | (13) |
| mTagBFP2-pBAD | Bacterial expression plasmid, Addgene item #54572 | (14) |
| pLJM1-PM-BFP | pLJM vector encoding mTagBFP2 fused to plasma membrane targeting sequence Rpre on its C-terminus | This study |
| pCMV-VSV-G | Vector encoding envelope protein for producing lentiviral and MuLV retroviral particles, Addgene item #8454 | (15) |
| psPAX2 | 2nd generation lentiviral packaging plasmid, Addgene item #12260 | Addgene |

**S4 Table. LABELS USED IN THIS STUDY.**

| ANTIBODY / NANOBODY | SOURCE | IDENTIFIER |
|---|---|---|
| Spot-label ATTO488, working dilution 1:4,000 – 1:5,000 | Chromotek/ Proteintech | Cat# eba488 |
| Spot-label ATTO594, working dilution 1:4,000 – 1:5,000 | Chromotek/ Proteintech | Cat# eba594 |
| ALFA-ATTO488, working dilution 1:500 | NanoTag Biotechnologies | Cat# N1502-At488-L |
| Anti-*Bordetella* Rabbit Serum, antigen *B. pertussis*, working dilution 1:500 – 1:1,000 | Dr. Vecerek, IMIC, Prague | Cat# N/A |
| Anti-Acetylated Tubulin antibody, working dilution 1:500 | Sigma-Aldrich/ Merck | Cat# T6793 |
| Anti-Rabbit IgG-DyLight 405, working dilution 1:500-1:600 | Jackson ImmunoResearch | Cat# 111-475-003 |
| Anti-Rabbit IgG-AF647, working dilution 1:600 | Jackson ImmunoResearch | Cat# 115-606-046 |
| Anti-Mouse IgG-AF568, working dilution 1:1,000 | ThermoFisher Scientific | Cat# A-11004 |
| Anti-Mouse IgG-AF488, working dilution 1:500 | Jackson ImmunoResearch | Cat# 115-546-062 |
| anti-ZO-1 Rabbit antibody, working dilution 1:300 | ThermoFisher Scientific | Cat# 339100 |
| WGA-AF647, wheat germ agglutinin conjugated with AF647, working dilution 5 µg/ml | Invitrogen/Thermo Fisher Scientific | Cat# 32466 |

**S5 Table. LIST OF qPCR PRIMERS USED IN THIS STUDY.**

| TARGET | NAME | SEQUENCE | AMPLICON SIZE (bp) | EFFICIENCY |
|---|---|---|---|---|
| *rpoB* | *rpoB*_for | acatgctgatcgcgttcatg | 118 | 91.99 |
| | *rpoB*_rev | acggtcagctcttcgatgtg | | |
| *dnaA* | *dnaA*_for | aggcgttcgacgatttcaag | 87 | 100.4 |
| | *dnaA*_rev | tgcgattcttgccggaaaag | | |
| *bsp22* | *bsp22*_for | accgtaaaggggatgctgg | 119 | 90.25 |
| | *bsp22*_rev | cgcttctcggtgttggtcat | | |
| *bscD* | *bscD*_for | gttcaaccagcgcaactacg | 95 | 101.53 |
| | *bscD*_rev | tcacgacactgcgtatctcg | | |

## REFERENCES


1. Simon R, Priefer U, Pühler A. 1983. A Broad Host Range Mobilization System for In Vivo Genetic Engineering: Transposon Mutagenesis in Gram Negative Bacteria. Bio/Technology 1:784.
2. Skopova K, Tomalova B, Kanchev I, Rossmann P, Svedova M, Adkins I, Bibova I, Tomala J, Masin J, Guiso N, Osicka R, Sedlacek R, Kovar M, Sebo P. 2017. Cyclic AMP-Elevating Capacity of Adenylate Cyclase Toxin-Hemolysin Is Sufficient for Lung Infection but Not for Full Virulence of Bordetella pertussis. Infect Immun 85.
3. Cotter PA, Miller JF. 1994. BvgAS-mediated signal transduction: analysis of phase-locked regulatory mutants of Bordetella bronchiseptica in a rabbit model. Infect Immun 62:3381-90.
4. Navarrete KM, Bumba L, Prudnikova T, Malcova I, Allsop TR, Sebo P, Kamanova J. 2023. BopN is a Gatekeeper of the Bordetella Type III Secretion System. Microbiol Spectr 11:e0411222.
5. Zmuda M, Sedlackova E, Pravdova B, Cizkova M, Dalecka M, Cerny O, Allsop TR, Grousl T, Malcova I, Kamanova J. 2024. The Bordetella effector protein BteA induces host cell death by disruption of calcium homeostasis. mBio doi:10.1128/mbio.01925-24:e0192524.
6. Bart MJ, Zeddeman A, van der Heide HG, Heuvelman K, van Gent M, Mooi FR. 2014. Complete Genome Sequences of Bordetella pertussis Isolates B1917 and B1920, Representing Two Predominant Global Lineages. Genome Announc 2.
7. Bart MJ, Harris SR, Advani A, Arakawa Y, Bottero D, Bouchez V, Cassiday PK, Chiang CS, Dalby T, Fry NK, Gaillard ME, van Gent M, Guiso N, Hallander HO, Harvill ET, He Q, van der Heide HG, Heuvelman K, Hozbor DF, Kamachi K, Karataev GI, Lan R, Lutynska A, Maharjan RP, Mertsola J, Miyamura T, Octavia S, Preston A, Quail MA, Sintchenko V, Stefanelli P, Tondella ML, Tsang RS, Xu Y, Yao SM, Zhang S, Parkhill J, Mooi FR. 2014. Global population structure and evolution of Bordetella pertussis and their relationship with vaccination. MBio 5:e01074.
8. Inatsuka CS, Xu Q, Vujkovic-Cvijin I, Wong S, Stibitz S, Miller JF, Cotter PA. 2010. Pertactin is required for Bordetella species to resist neutrophil-mediated clearance. Infect Immun 78:2901-9.
9. Posfai G, Kolisnychenko V, Bereczki Z, Blattner FR. 1999. Markerless gene replacement in Escherichia coli stimulated by a double-strand break in the chromosome. Nucleic Acids Res 27:4409-15.
10. Kovach ME, Phillips RW, Elzer PH, Roop RM, 2nd, Peterson KM. 1994. pBBR1MCS: a broad-host-range cloning vector. Biotechniques 16:800-2.
11. Kovach ME, Elzer PH, Hill DS, Robertson GT, Farris MA, Roop RM, 2nd, Peterson KM. 1995. Four new derivatives of the broad-host-range cloning vector pBBR1MCS, carrying different antibiotic-resistance cassettes. Gene 166:175-6.

12. Meisner J, Goldberg JB. 2016. The Escherichia coli rhaSR-PrhaBAD Inducible Promoter System Allows Tightly Controlled Gene Expression over a Wide Range in Pseudomonas aeruginosa. Appl Environ Microbiol 82:6715-6727.
13. Yeung T, Terebiznik M, Yu L, Silvius J, Abidi WM, Philips M, Levine T, Kapus A, Grinstein S. 2006. Receptor activation alters inner surface potential during phagocytosis. Science 313:347-51.
14. Subach OM, Cranfill PJ, Davidson MW, Verkhusha VV. 2011. An enhanced monomeric blue fluorescent protein with the high chemical stability of the chromophore. PLoS One 6:e28674.
15. Stewart SA, Dykxhoorn DM, Palliser D, Mizuno H, Yu EY, An DS, Sabatini DM, Chen IS, Hahn WC, Sharp PA, Weinberg RA, Novina CD. 2003. Lentivirus-delivered stable gene silencing by RNAi in primary cells. RNA 9:493-501.
